# THE REAL McCOIL: A method for the concurrent estimation of the complexity of infection and SNP allele frequency for malaria parasites

**DOI:** 10.1371/journal.pcbi.1005348

**Published:** 2017-01-26

**Authors:** Hsiao-Han Chang, Colin J. Worby, Adoke Yeka, Joaniter Nankabirwa, Moses R. Kamya, Sarah G. Staedke, Grant Dorsey, Maxwell Murphy, Daniel E. Neafsey, Anna E. Jeffreys, Christina Hubbart, Kirk A. Rockett, Roberto Amato, Dominic P. Kwiatkowski, Caroline O. Buckee, Bryan Greenhouse

**Affiliations:** 1 Center for Communicable Disease Dynamics, Department of Epidemiology, Harvard T.H. Chan School of Public Health, Boston, Massachusetts, United States; 2 Makerere University School of Public Health, College of Health Sciences, Kampala, Uganda; 3 Infectious Disease Research Collaboration, Kampala, Uganda; 4 Department of Medicine, Makerere University College of Health Sciences, Kampala, Uganda; 5 London School of Hygiene and Tropical Medicine, London, United Kingdom; 6 Department of Medicine, University of California, San Francisco, San Francisco, California, United States; 7 Genome Sequencing and Analysis Program, Broad Institute, Cambridge, Massachusetts, United States; 8 Wellcome Trust Centre for Human Genetics, University of Oxford, Oxford, United Kingdom; 9 Wellcome Trust Sanger Institute, Cambridge, United Kingdom; University of Chicago, UNITED STATES

## Abstract

As many malaria-endemic countries move towards elimination of *Plasmodium falciparum*, the most virulent human malaria parasite, effective tools for monitoring malaria epidemiology are urgent priorities. *P*. *falciparum* population genetic approaches offer promising tools for understanding transmission and spread of the disease, but a high prevalence of multi-clone or polygenomic infections can render estimation of even the most basic parameters, such as allele frequencies, challenging. A previous method, *COIL*, was developed to estimate complexity of infection (COI) from single nucleotide polymorphism (SNP) data, but relies on monogenomic infections to estimate allele frequencies or requires external allele frequency data which may not available. Estimates limited to monogenomic infections may not be representative, however, and when the average COI is high, they can be difficult or impossible to obtain. Therefore, we developed *THE REAL McCOIL*, Turning HEterozygous SNP data into Robust Estimates of ALelle frequency, via Markov chain Monte Carlo, and Complexity Of Infection using Likelihood, to incorporate polygenomic samples and simultaneously estimate allele frequency and COI. This approach was tested via simulations then applied to SNP data from cross-sectional surveys performed in three Ugandan sites with varying malaria transmission. We show that *THE REAL McCOIL* consistently outperforms *COIL* on simulated data, particularly when most infections are polygenomic. Using field data we show that, unlike with *COIL*, we can distinguish epidemiologically relevant differences in COI between and within these sites. Surprisingly, for example, we estimated high average COI in a peri-urban subregion with lower transmission intensity, suggesting that many of these cases were imported from surrounding regions with higher transmission intensity. *THE REAL McCOIL* therefore provides a robust tool for understanding the molecular epidemiology of malaria across transmission settings.

## Author Summary

Monitoring malaria epidemiology is critical for evaluating the impact of interventions and designing strategies for control and elimination. Population genetics has been used to inform malaria epidemiology, but it is limited by the fact that a fundamental metric needed for most analyses—the frequency of alleles in a population—is difficult to estimate from blood samples containing more than one genetically distinct parasite (polygenomic infections). A widely used approach has been to restrict analysis to monogenomic infections, which may represent a biased subset and potentially ignores a large amount of data. Therefore, we developed a new analytical approach that uses data from all infections to simultaneously estimate allele frequency and the number of distinct parasites within each infection. The method, called *THE REAL McCOIL*, was evaluated using simulations and was then applied to data from cross-sectional surveys performed in three regions of Uganda. Simulations demonstrated accurate performance, and analyses of samples from Uganda using *THE REAL McCOIL* revealed epidemiologically relevant differences within and between the three regions that previous methods could not. *THE REAL McCOIL* thus facilitates population genetic analysis when there are polygenomic infections, which are common in many malaria endemic areas.

This is a *PLOS Computational Biology* Methods Paper.

## Introduction

Malaria has declined significantly over the past decade, but continues to cause half a million deaths annually [1]. Calls for elimination have shifted research efforts towards developing new approaches for transmission reduction, including the identification of source and sink regions and hotspots that sustain transmission [2–4]. *Plasmodium falciparum* population genetic tools are increasingly being used to inform these efforts [5–12] and have been proposed as a means to establish the direction of parasite flows and to determine elimination status both by identifying the source of imported infections and by establishing that no local transmission is occurring [13–17]. However, in malaria-endemic regions, infections are frequently characterized by multiple different genotypes (polygenomic infections), which makes interpreting genetic data challenging. As a result, population genetic analyses of malaria parasites have often been limited to monogenomic infections, greatly reducing the utility of available data and potentially introducing biases into results.

Rapid technological developments have led to a proliferation of approaches for characterizing malaria parasite genomes, each with different implications for cost, suitability for field samples across a range of transmission settings, and applicability to different research questions [5,18–23]. Many genotyping approaches are based on a small number of single nucleotide polymorphisms (SNPs). SNP data are cheap and straightforward to obtain from commonly used dried blood spot (DBS) samples, collected in a variety of field settings, and remain the most common approach for genotyping studies. However, a high prevalence of polygenomic infections can render estimation of even the most basic parameters from SNP data, such as population allele frequencies, difficult.

Population allele frequencies are usually estimated from monogenomic infections [6,7,24], because of the challenge of estimating the true proportion of each lineage from heterozygous SNP loci resulting from high complexity of infection (COI, the number of clones in an individual). However, constraining data sets to only monogenomic infections may introduce systematic biases because these infections may not be representative. Such constraint also greatly limits the precision of estimates when the majority of samples are polygenomic. It is common to use the proportion of heterozygous calls in each individual or the fraction of polygenomic infections to compare genetic diversity between populations [6,7,16,25–27]. However, the complexity of infection underlying polygenomic infections can vary dramatically, and the probability of a particular locus being heterozygous will depend on its allele frequency in the population. *COIL* (estimating *COI* using likelihood), was recently developed to provide a more quantitative measure of genetic diversity [28], but unless supplied with external allele frequency data, relies on monogenomic infections to estimate allele frequencies and is therefore problematic when a large fraction of infections are polygenomic. While external allele frequency data can be obtained from parasite population genomic data such as the Pf3K project (http://www.malariagen.net/projects/pf3k*)*, these estimates are only available in specific locations, and may exhibit considerable heterogeneity in space and time.

Here we introduce a new Bayesian approach, Turning HEterozygous SNP data into Robust Estimates of ALelle frequency, via Markov chain Monte Carlo, and Complexity Of Infection using Likelihood (*THE REAL McCOIL)*, to additionally incorporate polygenomic samples, using Markov chain Monte Carlo methods to simultaneously estimate allele frequency and COI. We tested two versions of our method on a series of simulations and then applied it to data on 105 SNP loci in 868 samples from cross-sectional surveys in three regions of varying endemicity in Uganda [29–31]. The allele frequencies estimated by our new approach were used to calculate *F*_*ST*_ [32], a measure of genetic differentiation between sites, and *F*_*WS*_ [33]_,_ a measure of the within-host genetic diversity. These results demonstrate the utility of *THE REAL McCOIL* to obtain accurate estimates of COI and allele frequency from SNP data, which can be used to characterize genetic diversity and perform population genetic analyses of parasite populations even in very high transmission settings.

## Materials and methods

### Ethics statement

The cross sectional survey was approved by IRBs at the University of California, San Francisco (#11–07138) and SOMREC at Makerere University, Uganda (#2011–203).

### Methods to estimate population allele frequency and complexity of infection

We developed a Markov chain Monte Carlo (MCMC) method to simultaneously estimate population allele frequency for each SNP and COI for each individual. Since estimating COI and allele frequencies are highly related to each other, our approach explored the uncertainty of both at the same time, and by doing so, incorporated information from polygenomic infections. Assuming there are *n* individuals and *k* loci, the parameters to be estimated include complexity of infection for each individual (*M* = [*m*_*1*_, *m*_*2*_, …, *m*_*n*_]) and population allele frequency for each locus (*P =* [*p*_*1*_, *p*_*2*_, …, *p*_*k*_]). We used the data in two ways: a categorical method, in which we considered SNP at locus *j* of individual *i*, *B*_*ij*_, to be heterozygous or homozygous (0 [homozygous minor allele], 0.5 [heterozygous], 1 [homozygous major allele]), and a proportional method, in which the proportion of major allele at locus *j* of individual *i*, *S*_*ij*_, was calculated from the relative signal intensity for each allele ($S_{ij}=\frac{A_{1}{}_{ij}}{A_{1ij}+A_{2ij}}$, where *A*_*1*_ and *A*_*2*_ represent the signal intensity of major and minor allele that are obtained from Sequenom or similar types of SNP assays, respectively [34]). The notations are summarized in Table A in S1 File. Similar to *COIL*, *THE REAL McCOIL* assumed that different loci are independent, that different samples are independent and polygenomic infections are obtained from multiple independent infections, and that the samples were collected from a single homogeneous population.

#### Categorical method: Modeling heterozygous/homozygous calls

The likelihood of observing heterozygous/homozygous calls depends on COI, population allele frequency, and the probability of erroneously calling homozygous loci heterozygous (*e*_1_) and conversely calling heterozygous loci homozygous (*e*_2_). We have

$$L\left(M,\;P|B_{O}\right)=P\left(B_{O}|M,\;P\right)=\prod_{i=1}^{n}\prod_{j=1}^{k}\sum_{B_{T_{ij}}\in \left\{0,0.5,1\right\}}P\left(B_{Oij}|B_{Tij}\right)P\left(B_{Tij}|m_{i},\;p_{j}\right),$$ (1)

where *B*_*Tij*_ and *B*_*Oij*_ represent the true and observed heterozygosity at locus *j* of individual *i* (*B*_*Tij*_ and *B*_*Oij*_ ∈[0, 0.5, 1]). We specify *P*(*B*_*Oij*_|*B*_*Tij*_) to take the following form (Table 1), depending on the values of *B*_*Tij*_ and *B*_*Oij*_: and

$$P\left(B_{Tij}|m_{i},\;p_{j}\right)=\{\begin{array}{ccc} p_{j}{}^{m_{i}} & \text{if }B_{Tij}=1, & \\ {\left(1-p_{j}\right)}^{m_{i}} & \text{if }B_{Tij}=0, & \\ 1-p_{j}{}^{m_{i}}-{\left(1-p_{j}\right)}^{m_{i}} & \text{if }B_{Tij}=0.5.\; & \end{array}$$ (2)

**Table 1:** The observational model for categorical method.

		***B***_***Tij***_		
		**0**	**0.5**	**1**
***B***_***Oij***_	**0**	1−*e*_1_	*e*_2_/2	0
	**0.5**	*e*_1_	1−*e*_2_	*e*_1_
	**1**	0	*e*_2_/2	1−*e*_1_

We assumed uniform priors for *M* and *P* and updated them sequentially using a Metropolis-Hastings algorithm over *N* = 100,000 iterations, excluding the initial burn-in 1000 iterations to obtain the posterior distributions of *M* and *P*. If *e*_1_ and *e*_2_ were not pre-specified, *THE REAL McCOIL* estimated their posterior distributions along with *M* and *P*. The details of the sampling procedure are described in Text A in S1 File.

#### Proportional method: Modeling frequency data

The likelihood of obtaining the raw frequency of signals is composed of the observational model (*f*, the likelihood of observed frequency of signals given true within-host allele frequency) and the likelihood of true within-host allele frequency (*g*) as follows:

$$\begin{array}{l} L\left(M,P|S_{O}\right)=P\left(S_{O}\;|\;M,P\right)=\prod_{i=1}^{n}\prod_{j=1}^{k}P\left(S_{Oij}\;|\;m_{i},p_{j}\right)\\ =\prod_{i=1}^{n}\prod_{j=1}^{k}\left(\begin{array}{l} f\left(S_{Oij}\;|\;S_{Tij}=0\right)g\left(S_{Tij}=0\;|\;m_{i},p_{j}\right)\\ +\int_{0<S_{Tij}<1}f\left(S_{Oij}\;|\;S_{Tij}\right)g\left(S_{Tij}\;|\;m_{i},p_{j}\right)d_{S_{Tij}}\\ +f\left(S_{Oij}\;|\;S_{Tij}=1\right)g\left(S_{Tij}=1\;|\;m_{i},p_{j}\right) \end{array}\right) \end{array}$$ (3)

where *S*_*Tij*_ and *S*_*Oij*_ represent the true and observed frequency of major allele at locus *j* of individual *i* (0 ≤ *S*_*Tij*_, *S*_*Oij*_ ≤ 1). Consistent with other population genetic approaches [35], we assumed that each observation *S*_*Oij*_ was drawn from a normal distribution with the mean equal to the true frequency *S*_*Tij*_ and variance equal to $\sigma^{2}=\frac{\varepsilon_{est}}{\sqrt{A_{1ij}{}^{2}+A_{2ij}{}^{2}}}$, where *ε*_est_ represents the overall level of measurement error. The variance decreased with the intensity of the signal ($I=\sqrt{A_{1ij}{}^{2}+A_{2ij}{}^{2}}$). To exclude the values outside of [0, 1], we assumed point mass at 0 and 1 and their densities were obtained by integrating values from −∞ to 0 and from 1 to ∞, respectively.

That is,

$$f\left(S_{Oij}\;|\;S_{Tij}\right)=\{\begin{array}{ccc} \Phi \left(\frac{-S_{Tij}}{\sigma }\right)\; & \text{if }S_{Oij}=0\;\; & \\ \phi \left(\frac{S_{Oij}-S_{Tij}}{\sigma }\right)\; & \text{if }0<S_{Oij}<1 & \\ \Phi \left(\frac{S_{Tij}-1}{\sigma }\right)\; & \text{if }S_{Oij}=1\; & \end{array}$$ (4)

where Φ and *ϕ* are the cumulative distribution function and the probability density function of the standard normal distribution.

The density of the true within-host frequency was composed of a continuous distribution and point masses at 0 and 1 as follows:

$$g\left(S_{Tij}\;|\;m_{i},p_{j}\right)=\{\begin{array}{ll} {\left(1-p_{j}\right)}^{m_{i}} & \text{if }S_{Tij}=0\\ \left(1-p_{j}{}^{m_{i}}-{\left(1-p_{j}\right)}^{m_{i}}\right)\text{Beta}\left(S_{T_{ij}};\alpha_{m_{i}p_{j}},\beta_{m_{i}p_{j}}\right) & \text{if }0<S_{Tij}<1\\ p_{j}{}^{m_{i}} & \text{if }S_{Tij}=1 \end{array}$$ (5)

where $\text{Beta}\left(x;\alpha_{m_{i},p_{j}},\beta_{m_{i},p_{j}}\right)$ denotes the probability density function of the Beta distribution evaluated at *x*. The shape and scale parameters, $\alpha_{m_{i}p_{j}}$ and $\beta_{m_{i}p_{j}}$, respectively, depend on the complexity of infection (*m*_*i*_) and population allele frequency (*p*_*j*_), and were obtained by fitting the simulated data. We estimated values for $\alpha_{m_{i}p_{j}}$ and $\beta_{m_{i}p_{j}}$ pre-analysis, using simulated data to fit Beta distributions for a range of values for *m*_*i*_ and *p*_*j*_. To do this, we simulated the within-host allele frequency distribution for given values of *m*_*i*_ and *p*_*j*_ by sampling a single allele for each infection from a Bernoulli distribution with *p*_*j*_ and mixing these alleles with the relative contributions sampled from a uniform distribution as follows: sampling (*m*_*i*_ −1) numbers from a uniform distribution, ordering these numbers to obtain $u_{\left(1\right)},\;u_{\left(2\right)},\;\cdots ,\;u_{\left(m_{i}-1\right)}$, and mixing alleles using the proportions equal to the difference between them, $u_{\left(1\right)}-0,\;u_{\left(2\right)}-u_{\left(1\right)},\;\cdots ,\;u_{\left(m_{i}-1\right)}-u_{\left(m_{i}-2\right)},\;1-u_{\left(m_{i}-1\right)}$. Biologically, this means the proportion of either lineage can be any value between 0 and 1 with equal probability when *m*_*i*_ = 2. We then fit a Beta distribution to the resulting empirical distribution to obtain fitted values ${\hat{\alpha }}_{m_{i}p_{j}}$ and ${\hat{\beta }}_{m_{i}p_{j}}$. We performed this for each combination of *m* and *p*, where *m* ranged from 2 to 25 and *p* ranged from 0.01 to 0.99. As a continuous variable, we rounded observed values of *p* to the second decimal point to correspond to our discrete simulation range, selecting the appropriate $\alpha_{m_{i}p_{j}}$ and $\beta_{m_{i}p_{j}}$ to calculate the likelihood. Fig A in S1 File shows some examples of the distribution of simulated within-host allele frequencies with the fitted Beta distribution given *m* and *p*. While the fitted Beta parameters were obtained by simulating the ratio of mixing from a uniform distribution, the method performed well when the ratio of mixing was sampled from an exponential distribution, and *THE REAL McCOIL* can incorporate any fitted Beta distributions the users provide. We assumed uniform priors and updated *P*, *M*, *S*_*T*_ sequentially using a Metropolis-Hastings algorithm over *N* = 100,000 iterations, excluding the initial burn-in 1000 iterations to obtain posterior distributions of *P* and *M*. If *ε*_*est*_ was not pre-specified, *THE REAL McCOIL* estimated its posterior distribution along with *P* and *M*. The details of sampling procedure are described in Text A in S1 File.

### Simulations

We sampled COI of each individual from a zero-truncated Poisson distribution with mean $\bar{m}$, and population allele frequency of each locus from a uniform distribution *U*(*0*, *1*). For each individual, we independently sampled allele(s) for each locus from Bernoulli (*p*_*j*_). We determined the relative proportion of different lineages within the host by sampling the proportion of each infection from a uniform distribution *U*(*0*, *1*). For comparison, we additionally tried sampling from a truncated exponential distribution with the rate *λ* = 1. After obtaining within-host allele frequency (*S*_*Tij*_), we drew *S*_*Oij*_ from a normal distribution with mean = *S*_*Tij*_ and variance $\sigma^{2}=\frac{\varepsilon }{I}$, where *ε* represents the level of measurement error. We sampled the intensity of the signal *I* for each locus of each individual from the sum of a Poisson distribution with average $\bar{I}=8$ and a normal distribution with mean = 0 and variance = 0.25. Simulations were designed to represent the type of raw data obtained from Sequenom or similar types of SNP assays, where an intensity value is obtained for each potential allele [34]. If the intensity of signal was smaller than *I*_*min*_, we assumed the data were missing. We obtained the intensities of two alleles, *A*_*1*_ and *A*_*2*_, by $A_{1}=I\frac{S_{O}}{\sqrt{S_{O}{}^{2}+{\left(1-S_{O}\right)}^{2}}}$ and $A_{2}=I\frac{1-S_{O}}{\sqrt{S_{O}{}^{2}+{\left(1-S_{O}\right)}^{2}}}$, and determined heterozygous calls or homozygous calls by the relative intensity of signals of two alleles, which was characterized by $\text{arctan}\left(\frac{A_{1}}{A_{2}}\right)$, the angle in polar coordinate system. The SNP was called as heterozygous if $\text{arctan}\left(\frac{A_{1}}{A_{2}}\right)$ was within (*d*_1_, *d*_2_) and homozygous otherwise (Fig B in S1 File). For simulated data with measurement error *ε* >0, we used (*d*_1_, *d*_2_) = (5, 85). For real data, (*d*_1_, *d*_2_) was determined by expert review of each locus as described below.

We compared the performance of the categorical and proportional versions of our method to *COIL*, assessing the difference in parameter estimates and variation. We simulated violations of the model assumptions, specifically independence among loci, independence among parasite lineages within the same host, and a single, homogeneous population. Dependence among loci was simulated by different proportions of loci (*p*) that were linked. We simulated relatedness (*r*) among lineages within the same host by sampling alleles either from an existing lineage within the same host (with probability *r*) or from the population (with probability (1-*r*)). We simulated two equally sized subpopulations with either the same or different average COI and with various levels of difference in allele frequencies and treated them as one single population to test the robustness of the assumption that the population was well-mixed. We also simulated missing data and populations with COI up to 20.

### Genotyping of field samples

Dried blood spot samples were obtained from representative cross-sectional surveys performed in 2012 and 2013 as part of the East African International Centers of Excellence in Malaria Research (ICEMR) program. Surveys were performed in each of three sub-counties in Uganda: Nagongera in Tororo District, Kihihi in Kanungu District, and Walukuba in Jinja District. Details of these surveys, along with entomological and cohort data from the same sites have been published [29,31,36,37]. In brief, 200 households from each sub-county were randomly selected from a census population, and all children and an age-stratified sample of adults were enrolled from each household. All samples taken from individuals with evidence of asexual parasitemia by microscopy were selected for Sequenom SNP genotyping, and an age-stratified subset were also selected for merozoite surface protein 2 (*msp2*) genotyping. The Sequenom assay consisted of 128 SNPs selected to be polymorphic and at intermediate/high frequency in multiple popluations (https://www.malariagen.net/projects/p-falciparum-community-project). After removing variants with elevated missing rate, we retained 105 SNPs (see S1 Table for SNP data) and three of them are in known drug resistance loci. Samples were genotyped according to the relative intensity of the two alleles, as previously described [21]. Genotyping of *msp2* was performed with alleles sized by capillary electrophoresis, as previously described [38]. The number of unique alleles were called by a single, expert reader, with allele counts > 5 grouped into a single category due to difficulties in accurately distinguishing artifacts from true alleles at high complexities of infection.

### Data analysis

After excluding samples with more than 25% missing SNP data and loci with more than 20% missing data from the analysis, the numbers of individuals included were 462 (71%) [Nagongera], 48 (51%) [Walukuba], and 74 (59%) [Kihihi], and the numbers of loci were 63 (60%) [Nagongera], 49 (47%) [Walukuba], and 52 (50%) [Kihihi]. After these cutoffs, only the analysis of Nagongera included one drug resistance locus, and others included none. We used a permutation test with *N* = 10,000 to compare estimated COI between groups because there were many ties. In the analysis, we assumed that error rates *e*_1_ and *e*_2_ were both 0.05 and *ε*_*est*_ = 0.02. *F*_*WS*_ was calculated by (1−*H*_*W*_/*H*_*S*_), where *H*_*W*_ and *H*_*S*_ are 2*p*_*W*_(1−*p*_*W*_) and 2*p*_*S*_(1−*p*_*S*_) respectively and *p*_*w*_ and *p*_*s*_ are within-host allele frequency and population allele frequency respectively [33]. The *H*_*W*_/*H*_*S*_ ratio was estimated by performing linear regression between *H*_*W*_ and *H*_*S*_ with fixed intercept = 0.

## Results

### Simultaneously estimating allele frequencies and the complexity of infection

**Fig 1:**
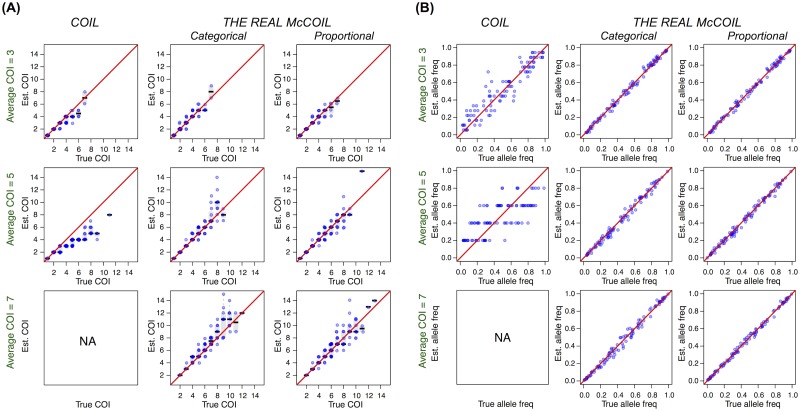
True vs. estimated values of COI (A) and allele frequencies (B) using *COIL* and *THE REAL McCOIL*. Each blue dot represents a sample. The black bar and the grey box show the median and 25% to 75% quantile. *THE REAL McCOIL* estimated allele frequencies and COI better than *COIL*, especially when the average COI was high and the majority of infections were polygenomic.

We simulated data of 100 SNPs from populations with an average COI of 3, 5 and 7 and sample size of 100, and compared estimates of COI and allele frequencies using *COIL* and *THE REAL McCOIL*. When average COI was 3, all three methods estimated COI well, although allele frequency estimates from *COIL* were less precise than *THE REAL McCOIL* (mean absolute deviation [MAD] = 0.077 [*COIL*], 0.019 [*THE REAL McCOIL categorical*], 0.019 [*THE REAL McCOIL proportional*], Mann-Whitney test *p*-value < 2×10^−16^) (Fig 1). When average COI was 5, however, *COIL* did not estimate COI or allele frequencies accurately (MAD = 1.45 [COI] and 0.15 [allele frequency]), and when COI was 7, it was unable to estimate allele frequencies due to a lack of monogenomic infections. In contrast to *COIL*, which consistently underestimated or failed to estimate COI in populations with greater numbers of polygenomic infections, *THE REAL McCOIL* estimated both COI and allele frequencies well even when COI was high (for categorical and proportional methods, respectively: COI = 5, MAD = 0.61, 0.45 [COI] and 0.024, 0.019 [allele frequency]; COI = 7, MAD = 0.86, 0.79 [COI] and 0.025, 0.015 [allele frequency]). Thus, the ability of *THE REAL McCOIL* to jointly estimate allele frequencies and COI from all available data resulted in considerably improved performance in estimates of both quantities, especially when the average COI was high.

Furthermore, we compared the performance of the categorical and proportional methods when we included measurement error in simulations of observed within-host allele frequency. The categorical method modeled measurement error by incorporating the probability of calling homozygous loci heterozygous (*e*_1_) and vice versa (*e*_2_) in the likelihood equation, and the proportional method modeled measurement error by assuming that the difference between true and observed within-host allele frequencies decreased with the intensity of the signals, and was proportional to the error parameter (*ε*_*est*_). Fig C (A)(C) in S1 File shows that measurement error resulted in a systematic bias in estimates of COI. However, this bias was relatively minor and fairly robust to misspecification of measurement error, especially when the proportional method was used. In addition, allele frequencies were accurately estimated by both methods (Fig C (B)(E) in S1 File). If parameters for measurement error were not specified, *THE REAL McCOIL* fit them as part of the MCMC. Fig C (D)(F) in S1 File shows that the probability that the 95% credible interval contained the true COI when error parameters were fitted was higher than those when error parameters were greatly mis-specified.

### Sensitivity analysis

We next simulated specific violations of the model assumptions to test the robustness of our approach. In particular, we examined the impact of linkage disequilibrium between loci, genetic relatedness of parasites within an individual host, and relatedness between subsets of individuals within the overall population (population substructure). When a proportion of loci (*p*) were completely linked, COI was slightly overestimated (Fig D in S1 File). When different lineages in the same host were not independent, COI was underestimated and the level of underestimation of COI increased with the level of relatedness (*r*) (Fig E in S1 File). When we treated two subpopulations as one population, COI was underestimated and the difference between true and estimated COI increased with the difference in the average of COI and the difference in allele frequencies between two subpopulations (Fig F in S1 File). Of these three violations of model assumptions, only a high degree of relatedness between parasites within an individual host resulted in substantial bias in estimates of COI, and none substantially affected estimates of population allele frequencies. Genotyping of real samples often results in missing data; both methods performed well even when 50% of the data were missing (Fig G in S1 File). Furthermore, we tested how the number of loci influences the performance of estimating COI. While the probability that 95% credible interval contained the true COI did not change with the number of loci, the average difference between true and estimated COI decreased (Fig H in S1 File). *THE REAL McCOIL* provided unbiased estimates even when COI was very high (e.g. 15–20), despite the uncertainty of the estimates increasing with true COI (Fig I in S1 File).

### Complexity of infection and allele frequencies in three regions of Uganda

We next applied *THE REAL McCOIL* to data on 105 SNPs generated from smear positive individuals identified in cross-sectional surveys in three regions of Uganda [36,37] and compared results obtained from *THE REAL McCOIL* to those using *COIL*. Both categorical and proportional methods were applied and showed consistent results; for simplicity we therefore present only results from the categorical method.

**Fig 2:**
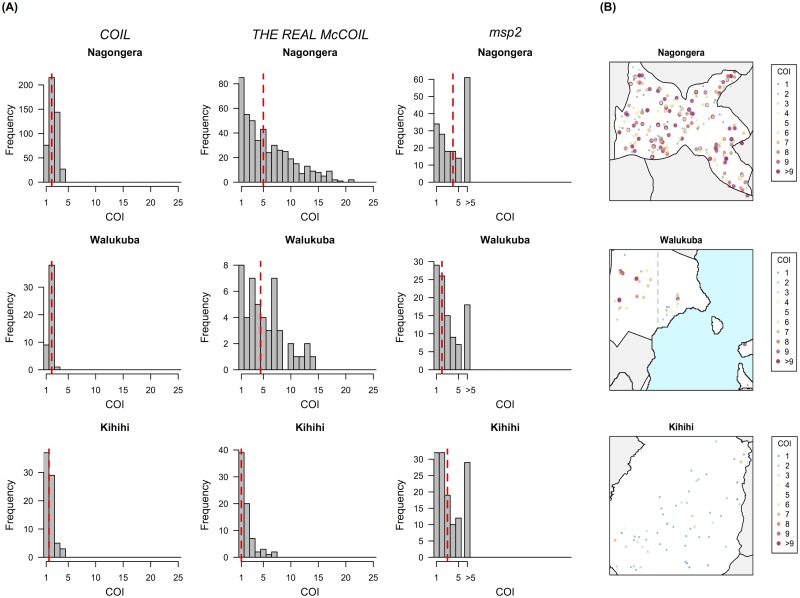
Estimates of COI in Nagongera, Walukuba, and Kihihi. **(A)** Estimates of COI by *COIL*, *THE REAL McCOIL*, and *msp2*. For *THE REAL McCOIL*, the point estimates of COI shown are medians from the posterior distributions. The COI estimated by *THE REAL McCOIL* in Nagongera and Walukuba were similar, and much higher than that in Kihihi (median COI = 5 [Nagongera], 4.5 [Walukuba], and 1 [Kihihi]; permutation test, *p*-values = 0.158 [Nagongera vs. Walukuba], 0.002 [Nagongera vs. Kihihi], 0.0006 [Walukuba vs. Kihihi]). Allele counts > 5 in *msp2* typing were grouped into a single category due to difficulties in accurately distinguishing artifacts from true alleles at high complexities of infection. The dashed red lines represent the medians of COI in three regions. **(B)** The spatial distribution of estimated COI by *THE REAL McCOIL* in three regions. Small random noise was added to the location of samples in the map. COI of samples collected from the West of Walukuba was higher than those from the East of Walukuba (medians = 5 [West] and 3 [East], *p*-value = 0.027).

Nagongera, Kihihi, and Walukuba have been shown to have transmission intensities varying by approximately 100 fold, with entomological inoculation rates recently measured at 310, 32, and 2.8 infectious bites per person year, respectively [29]. Using *COIL*, the estimated COI was relatively low, with little difference between the 3 sites (median COI = 2 [Nagongera], 2 [Walukuba], and 1.5 [Kihihi]) (Fig 2A). In contrast, results from *THE REAL McCOIL* show that the COI in Nagongera and Walukuba were similar, and much higher than that in Kihihi (median COI = 5 [Nagongera], 4.5 [Walukuba], and 1 [Kihihi])(Fig 2A, Table B in S1 File and S2 Table). These differences between sites were not captured by *COIL* because of its dependence on monogenomic infections to obtain estimates of allele frequencies, which were rare in these individuals. We also compared our results to COI estimated using another standard method, *msp2* typing, which was performed on a subset of the samples (Fig J in S1 File). Unlike *THE REAL McCOIL*, however, *msp2* typing estimated similar COI in Walukuba and Kihihi (*p*-value = 0.49) (Fig 2A). *msp2* encodes an antigen that elicits strong antibody responses, and this discrepancy may be due to complex population structure arising from immune selection. The difference may also result from the resolution of *msp2* typing, which is constrained to COI ≤ 5 [39], or the fact that it is a single marker, rather than a collection of genome-wide markers.

**Fig 3:**
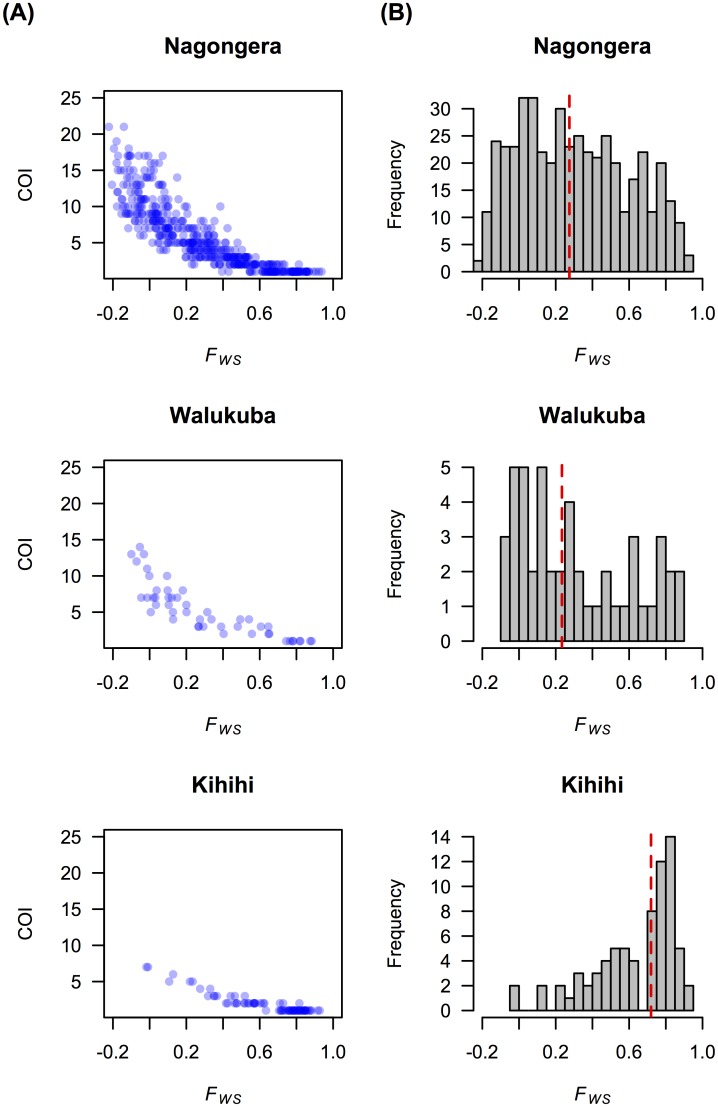
*F*_*WS*_. **(A)** Estimated COI by *THE REAL McCOIL* was negatively associated with *F*_*WS*_. **(B)** *F*_*WS*_ in Kihihi was higher than Nagongera and Walukuba. The *F*_*WS*_ values shown were calculated using population allele frequencies estimated from categorical method of *THE REAL McCOIL*.

The high COI observed in the lowest transmission site of Walukuba was unexpected but reflected clear differences in the proportion of heterozygous calls, which was similar between Nagongera and Walukuba and lower in Kihihi (Fig K in S1 File). The distributions of age and parasite density were similar between the sites, and thus unlikely to explain these differences (Fig L and Fig M in S1 File). We calculated *F*_*WS*,_ an inverse measure of outcrossing [33,40], and found that it was significantly negatively associated with our COI estimates (Fig 3; Pearson’s correlation test between log(COI) and *F*_*WS*_, *ρ* = −0.93 [Nagongera], −0.94 [Walukuba], and −0.95 [Kihihi], *p*-values < 2.2×10^−16^ for all). *F*_*WS*_ in Nagongera and Walukuba are similar and lower than that in Kihihi, suggesting that the level of outcrossing is smallest in Kihihi, which is consistent with the pattern of COI.

We also examined the relationship between COI and epidemiological and geographical factors within each site. In Nagongera, COI in young children increased with age until peaking at age 7, and then decreased; sample sizes for the other two sites were too small to estimate trends (Fig N in S1 File). Interestingly, parasite density was negatively correlated with COI after adjusting for age (partial correlation *r* = −0.15 [Nagongera], −0.27 [Walukuba], −0.23 [Kihihi], *p*-values = 0.0011 [Nagongera], 0.058 [Walukuba], 0.043 [Kihihi]). This negative association was most pronounced in those aged 3–10 years in Nagongera (Fig O in S1 File), and may reflect the dominance of particular clones in acute, high-density infections. No differences in COI were observed between households with or without Insecticide Treated Nets (ITNs), or between sampling years.

In Kihihi, elevation and COI were negatively associated (*r* = −0.259, *p*-value = 0.026), consistent with the previously identified negative associations between elevation and mosquito density, the incidence of malaria, and serological evidence of exposure [41]. Interestingly, the unexpectedly high COI observed in Walukuba was largely driven by samples collected from the West of this sub-county, (Fig 2B; medians = 5 [West] and 3 [East], *p*-value = 0.027). We have previously noted that mosquito densities in Walukuba are lower in the West, which is closer to urban centers, as compared to the East, which is a fishing village comprised largely of makeshift wooden housing [42]. One potential explanation for this seemingly paradoxical finding—high COI in the lowest transmission part of the lowest transmission site–is that a substantial proportion of these infections were imported from areas of higher transmission, where parasite populations are more diverse and co-transmission of multiple genetically distinct parasites is more likely.

Finally, we compared allele frequencies from each of the three sites to determine whether there was any evidence of population differentiation. We found little genetic differentiation between sites measured based on our estimated allele frequencies (*F*_*ST*_ ranged from 0.004 to 0.04; Table C in S1 File and S3 Table), although Kihihi, which is somewhat geographically isolated, had slightly higher *F*_*ST*_ with respect to the other two sites.

## Discussion

Despite the availability of increasingly efficient genotyping technologies for molecular epidemiology, the prevalence of polygenomic infections in malaria-endemic regions hinders the estimation of basic population genetic parameters for *Plasmodium falciparum*. While *COIL* can estimate COI using allele frequencies from monogenomic infections or external data, direct estimation of allele frequencies from all samples is a preferable approach, particularly when no relevant frequency data are available and sample size is sufficient to overcome stochastic sampling error. *THE REAL McCOIL* accomplishes this by incorporating information from polygenomic infections to simultaneously estimate COI and population allele frequencies. We show through detailed simulations that our approach is robust to most model assumptions and can readily handle missing data. In addition, *THE REAL McCOIL* can utilize raw SNP genotyping data, allowing the method to be robust to errors in allele calling. Analysis of genotyping data from Uganda show that *THE REAL McCOIL* is able to identify nuances in field data that previous methods could not. In particular, compared with *msp2* genotyping or applying *COIL* to SNP data, we identified much higher average COI overall and epidemiologically relevant variation between and within study sites.

Through a number of simulations, we show that results obtained from *THE REAL McCOIL* are robust to assumptions that loci are independent and that the parasite population is homogeneous. As would be expected, a high degree of relatedness between parasites within an individual host resulted in substantial downward bias in estimates of COI. This is not trivial, as parasites in some epidemiological settings may be closely related within a host, e.g. due to co-transmission [43]. Fortunately, we found that this bias follows a clear linear pattern and can either be corrected if the level of relatedness is known, estimated directly from the data, or can at least be given reasonable bounds (Text B in S1 File). While estimating the level of relatedness may be challenging, enough information may be present in the data to do so in some cases, as demonstrated by a recent paper which estimated this parameter from sequence-read data [44]. *THE REAL McCOIL* can also be applied to read-based SNP data, and in theory can be extended to estimate relatedness. While we note that the most obvious model for measurement error in sequence-read data is a binomial distribution (Text C in S1 File), a normal distribution as applied in our current version offers a reasonable approximation and has computational advantages.

Genotyping of one or a few highly polymorphic antigen markers, such as *msp1* and *msp2*, is currently the most common method for determining COI [45,46]. The use of capillary electrophoresis has improved resolution of alleles, but due to the creation of PCR artifacts it is still difficult to accurately measure COI > 5 [38]. Deep sequencing of antigens such as *csp* is an alternative approach [47,48]. However, with all of these approaches, immune selection on these genes within individuals and in a population can bias estimates of COI in ways which are difficult to predict [49,50]. Since loci under different types of selection can evolve independently in the presence of recombination, the diversity and geographic distribution of loci under immune selection may not be the same as observed among SNP loci. Both recombination rate and immune selection pressure will vary systematically with transmission intensity, resulting in complex associations between different genetic markers. Therefore, multiple genetic lineages defined by SNP panels may be associated with few *msp2* alleles, or vice versa, depending on the transmission setting and selective environment. In addition, if lineages within the host are related, using multiple markers across the genome is more likely to detect multiple lineages than using one region of the genome. *F*_*WS*_, based on the difference between within-host and population heterozygosity, is a related metric used to quantify within-host diversity [33]. While *F*_*WS*_ is correlated with COI, the metric is conceptually different because it is influenced by both the relative proportions of lineages within the host and population allele frequencies [21,33,40]. *estMOI* [51] uses phasing information from sequence reads and the number of unique allelic combinations to estimate COI but requires deep sequencing data and can be biased by sequencing error. Some methods that use SNP data to estimate haplotype frequencies also simultaneously estimate COI [52,53]. However, current haplotype-based methods can only consider a limited number of loci (~7) because the number of possible haplotypes quickly expands with the number of loci. We expect that *THE REAL McCOIL* is better at estimating COI than these methods because it can incorporate a much larger number of SNPs. Moreover, COI estimated from *THE REAL McCOIL* could be used as a prior in tools estimating haplotype frequencies.

Application of *THE REAL McCOIL* to genotyping data from Uganda allowed us to calculate allele frequencies and *F*_*ST*_, which was not possible to do from the raw data or using *COIL* due to the high proportion of heterozygous calls. *THE REAL McCOIL* also provided estimates of COI for all sites, which demonstrated associations with epidemiologic factors not identified using *msp2* genotyping. Interestingly, we identified a high COI in the lowest transmission site, potentially indicating importation of parasites from higher transmission areas. Although the possibility remains that recent transmission reduction left complex, chronic infections in its wake, explaining the high COI observed in Walukuba, the simplest explanation is that these infections were imported from high transmission settings nearby. Additionally, our results demonstrated that COI increased with age until age 7, and subsequently decreased, consistent with studies based on *msp1* and/or *msp2* typing [54–59]. Previous studies reported inconsistent associations between COI and parasite density for children *>* 2 years old (positive [55,58,60], none [54,61], or negative [62]). We observed a negative association between COI and parasite density in children aged 3–10 in Nagongera. Although higher parasite density may help detect more strains within the host [63–65], the detection of minority strains may be more influenced by relative proportions of the strains [39]. Individuals with high parasite densities may be relatively immunologically naïve and have one or few lineages dominating the infection [66]. Lower parasite densities may be associated with partial immunity and parasite persistence, and consequently the accumulation of parasite lineages [67–71]. Also, parasite lineages are more likely to persist and accumulate in people with low parasite density because they are less likely to have clinical symptoms [70,72] and be treated. The discrepancy between studies can be due to different genetic markers, different transmission setting and immune levels, different contribution of co-transmission vs. superinfections, or some combination of these factors.

In summary, *THE REAL McCOIL* facilitates population genetic analysis of SNP data from polygenomic infections, which are common in many transmission settings and may predominate even in low transmission settings. Population allele frequency, which was previously difficult to estimate if the majority of samples were polygenomic, can be estimated by *THE REAL McCOIL*, allowing downstream analysis that requires frequencies, such as estimating *F*_*ST*_, *F*_*WS*,_ and effective population size (*N*_*e*_) [32,33,73]. *THE REAL McCOIL* is not only limited to *P*. *falciparum*, but can also be applied to other parasite species with polygenomic infections [74], including *Plasmodium vivax* [75]. Codes for *THE REAL McCOIL* are available on GitHub (https://github.com/Greenhouse-Lab/THEREALMcCOIL).

## Supporting information

S1 File Supporting information. Supplementary texts, figures and tables.
(PDF)

S1 Table SNP data. (TXT)

S2 Table The 95% credible intervals of COI of samples from Uganda. (TXT)

S3 Table The 95% credible intervals of allele frequencies. (TXT)
