# Supplementary material for: THE REAL McCOIL: A method for the concurrent estimation of the complexity of infection and SNP allele frequency for malaria parasites
Source: PLoS Comput Biol. 2017 Jan 26;13(1):e1005348. doi: 10.1371/journal.pcbi.1005348 (PMC5300274; doi:10.1371/journal.pcbi.1005348)
Supplement: S1 File — Supplementary texts, figures and tables. (PDF) [file pcbi.1005348.s001.pdf]

S1 File. Supplementary materials

Text A. Supplementary method

Sampling procedure for categorical method

A Metropolis–Hastings algorithm was used to sample from the posterior distribution for each parameter. Our prior distribution for COI values was a discrete uniform distribution ranging from 1 to m_{max}, while for allele frequencies we chose a uniform prior distribution on the unit interval [0, 1]. We started with initial COI equal to 1 and initial population allele frequencies calculated from the numbers of homozygous calls ($N_{B_{Oij}=1}$ and $N_{B_{Oij}=0}$) and heterozygous calls ($N_{B_{Oij}=0.5}$) with the assumption that within-host allele frequency of heterozygous calls was 0.5 as follows:

$$\text{initial } p_j = \frac{N_{B_{Oij}=1} + 0.5N_{B_{Oij}=0.5}}{N_{B_{Oij}=1} + N_{B_{Oij}=0} + N_{B_{Oij}=0.5}}. \quad \text{(s1)}$$

1. We proposed an update for each m_i, $m_i^* = m_i \pm 1$ with equal probabilities. Proposed values $m_i^* > m_{max}$ or $m_i^* < 1$ fell outside the support of our prior and were rejected; otherwise, we accepted m_i^* with the probability

$$= \min\left\{1, \frac{q(m_i \mid m_i^*)L(m_i^*)}{q(m_i^* \mid m_i)L(m_i)}\right\} = \min\left\{1, \frac{L(m_i^*)}{L(m_i)}\right\} \quad \text{(s2)}$$

, where *q()* denotes the proposal distribution, and $q(m_i \mid m_i^*)$ = $q(m_i^* \mid m_i)$ =0.5 due to symmetry. *P* and other elements in *M* from the previous step were used for calculating $L(m_i^*)$ and $L(m_i)$.

2. We proposed an update for each p_j, $p_j^* \sim N(p_j, \sigma_p^2)$, where σ_p^2 =0.1. Proposed values p_j^* outside the unit interval were rejected; otherwise, we accepted p_j^* with the probability

$$= \min\left\{1, \frac{q(p_j \mid p_j^*)L(p_j^*)}{q(p_j^* \mid p_j)L(p_j)}\right\} = \min\left\{1, \frac{L(p_j^*)}{L(p_j)}\right\} \quad \text{(s3)}$$

, where $q(p_i \mid p_i^*)$ and $q(p_i^* \mid p_i)$ cancel due to symmetry of normal distribution. *M* and other elements in *P* from the previous step were used when calculating $L(p_j^*)$ and $L(p_j)$.

Steps 1 and 2 were repeated *N* times. The posterior distribution of each parameter was obtained by the distribution of parameters in the Markov chain after an initial burn-in of 1000 iterations was discarded.

If the parameters of measurement error, e_1 and e_2, were not pre-specified, they were estimated with COI and allele frequencies. After step 2, we added an additional step to update e_1 and e_2 as follows:

3. We proposed an update for e_1, $e_1^* \sim N(e_1, \sigma_e^2)$, where σ_e^2 =0.0001. Proposed values e_1^* outside the unit interval were rejected; otherwise, we accepted e_1^* with the probability

$$= \min\left\{1, \frac{q(e_1 \mid e_1^*)L(e_1^*)}{q(e_1^* \mid e_1)L(e_1)}\right\} = \min\left\{1, \frac{L(e_1^*)}{L(e_1)}\right\} \quad \text{(s4)}$$

, where $q(e_1 \mid e_1^*)$ and $q(e_1^* \mid e_1)$ cancel due to symmetry of normal distribution. M, P, and e_2 from the previous step were used when calculating $L(e_1^*)$ and $L(e_1)$. We then updated e_2 using the same method as updating e_1.

Sampling procedure for proportional method

We updated M, P, and S_T sequentially using a Metropolis-Hastings algorithm. We assumed a uniform prior on the unit interval for S_T; priors for COI and allele frequencies were as before. We started with the initial values of M, P, and S_T. As with the categorical method, the initial values for M were 1 and the initial values for P were calculated by assuming that within-host allele frequency of heterozygous calls is 0.5. The initial values for S_T were equal to S_0.

1. We proposed an update for each m_i, m_i^*, and accepted or rejected m_i^* using the same approach as in the categorical method. P, S_T, and other elements in M from the previous step were used to calculate $L(m_i^*)$ and $L(m_i)$.

2. We proposed an update for each p_j, p_j^*, and accepted or rejected p_j^* using the same approach as in the categorical method. M, S_T, and other elements in P from the previous step were used for calculating $L(p_j^*)$ and $L(p_j)$.

3. We proposed an update of each S_{Tij}, $S_{Tij}^* \sim N(S_{Tij}, \sigma_s^2)$, where $\sigma_s^2=0.1$. If S_{Tij}^* was smaller than 0 or greater than 1, we set S_{Tij}^* to 0 or 1, respectively. We accepted S_{Tij}^* with the probability

$$= \min\left\{1, \frac{q(S_{Tij} \mid S_{Tij}^*) L(S_{Tij}^*)}{q(S_{Tij}^* \mid S_{Tij}) L(S_{Tij})}\right\} \tag{s5}$$

, where

$$q(a \mid b) = \begin{cases} \Phi\left(\frac{-b}{\sigma_s}\right) & \text{if } a = 0 \\ \Phi\left(\frac{b-1}{\sigma_s}\right) & \text{if } a = 1 \\ \phi\left(\frac{a-b}{\sigma_s}\right) & \text{if } 0 < a < 1 \end{cases} \tag{s6}$$

M, P, and other elements in S_T from the previous step were used to calculate $L(S_{Tij}^*)$ and $L(S_{Tij})$.

Steps 1 to 3 were repeated N times. The posterior distribution of each parameter was obtained by the distribution of parameters in the Markov chain after an initial burn-in of 1000 iterations was discarded.

If the parameter of measurement error, ε_{est}, was not pre-specified, it was estimated with COI and allele frequencies. After step 3, we added an additional step to update ε_{est} as follows:

3. We proposed an update for ε_{est}, $\varepsilon_{est}^* \sim N(\varepsilon_{est}, \sigma_e^2)$, where $\sigma_e^2 = 0.0001$. Proposed values ε_{est}^* outside the unit interval were rejected; otherwise, we accepted ε_{est}^* with the probability

$$= \min\left\{1, \frac{q(\varepsilon_{est} \mid \varepsilon_{est}^*) L(\varepsilon_{est}^*)}{q(\varepsilon_{est}^* \mid \varepsilon_{est}) L(\varepsilon_{est})}\right\} = \min\left\{1, \frac{L(\varepsilon_{est}^*)}{L(\varepsilon_{est})}\right\} \tag{s7}$$

, where $q(\varepsilon_{est} \mid \varepsilon_{est}^*)$ and $q(\varepsilon_{est}^* \mid \varepsilon_{est})$ cancel due to symmetry of normal distribution. M, P, and S_T from the previous step were used when calculating $L(\varepsilon_{est}^*)$ and $L(\varepsilon_{est})$.

Text B. Correcting for relatedness

Fig E in S1 File shows that there is a linear relationship between estimated and true COI and the slope is very close to (1−*r*). If *r* is known, we can correct for the estimates of COI using the fitted regression model obtained from simulations. Specifically, if the fitted model is $COI_{est} = \beta_0 + \beta_1 COI_{true}$ for the known value of *r*, we can obtain COI_{true} by $(COI_{est} - \beta_0)/\beta_1$. The results from our simulations suggest that β_1 can be approximated by (1−*r*).

Text C. Proposed proportional method: Modeling sequence-read data

Same as the mass spectrometry-based data, the likelihood of obtaining the raw frequency of sequence reads is composed of the observational model and the likelihood of true within-host allele frequency (g) (equation (3)). The likelihood of true within-host allele frequency (g) is the same as the original method (equation (5)). A more suitable observational model for sequence-read data is binomial distribution because sequence-read data are discrete rather than continuous. The observational model based on binomial distribution is as follows:

$$f\left(S_{Oij} \mid S_{Tij}\right) = \begin{cases} Binom\left(R_{ij}S_{Oij} \middle| R_{ij}, e_3\right) & \text{if } S_{Tij} = 0 \\ \sum_{k=0}^{R_{ij}} Binom\left(k \middle| R_{ij}, S_{Tij}\right)\left(\sum_{l=\max\{0, R_{ij}S_{Oij}-R_{ij}+k\}}^{\min\{k, R_{ij}S_{Oij}\}} Binom\left(l \middle| k, 1-e_3\right) Binom\left(R_{ij}S_{Oij} - l \middle| R_{ij} - k, e_3\right)\right) & \text{if } 0 < S_{Tij} < 1 \\ Binom\left(R_{ij}\left(1 - S_{Oij}\right) \middle| R_{ij}, e_3\right) & \text{if } S_{Tij} = 1 \end{cases} \quad \text{(s8)}$$

, where R_{ij} and S_{Oij} represents the total number of reads and the proportion of reads showing major allele at locus j of individual i, e_3 is the probability of sequencing error, and *Binom* is the probability mass function of the binomial distribution. The observed reads showing major allele can come from either the presence of major allele within the host or sequencing error. However, because the computational time is much higher than using normal distribution for the observational model, at this time this is not implemented in *THE REAL McCOIL*.

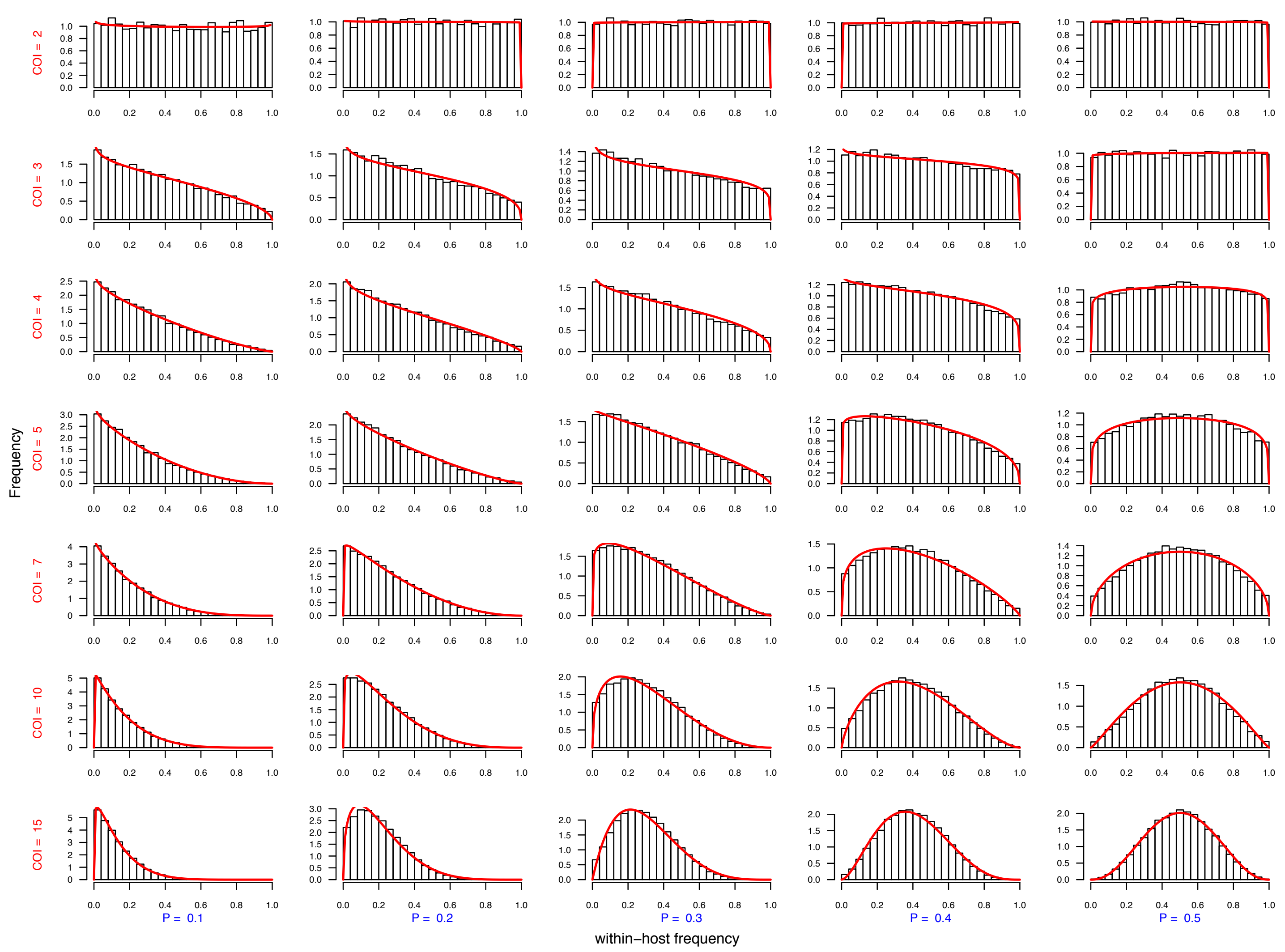

Fig A. Simulated within-host allele frequencies given different COI and population allele frequencies and the fitted Beta distributions. We simulated the within-host allele frequency distribution for given values of m_i and p_j by sampling a single allele for each infection from a Bernoulli distribution with p_j and mixing these alleles with the relative contributions sampled from a uniform distribution as follows: sampling $(m_i - 1)$ numbers from a uniform distribution, ordering these numbers to obtain $u_{(1)}, u_{(2)}, \cdots, u_{(m_i-1)}$, and mixing alleles using the proportions equal to the difference between them, $u_{(1)} - 0, u_{(2)} - u_{(1)}, \cdots, u_{(m_i-1)} - u_{(m_i-2)}, 1 - u_{(m_i-1)}$. We fit a Beta distribution to the resulting empirical distribution to obtain fitted values $\hat{\alpha}_{m_i p_j}$ and $\hat{\beta}_{m_i p_j}$. The fitted Beta distributions are shown in red. For this particular way of mixing alleles, there is an analytical solution for the within-host allele frequencies, which is a binomial mixture of Beta distributions. *THE REAL McCOIL* can incorporate any fitted Beta distributions users provide.

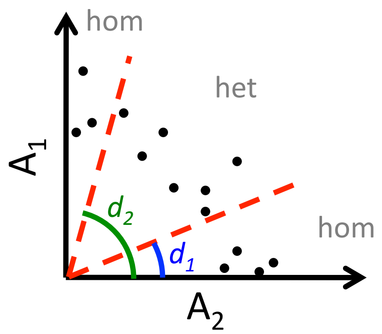

Fig B. Heterozygous or homozygous calls were determined by relative signals of two alleles. Relative signals of two alleles were characterized by arctan(A_1/A_2). If $\arctan\left(\frac{A_1}{A_2}\right) \leq d_1$, $B_{0ij}=0$; if $\arctan\left(\frac{A_1}{A_2}\right) \geq d_2$, $B_{0ij}=1$; if $d_1 < \arctan\left(\frac{A_1}{A_2}\right) < d_2$, $B_{0ij}=0.5$.

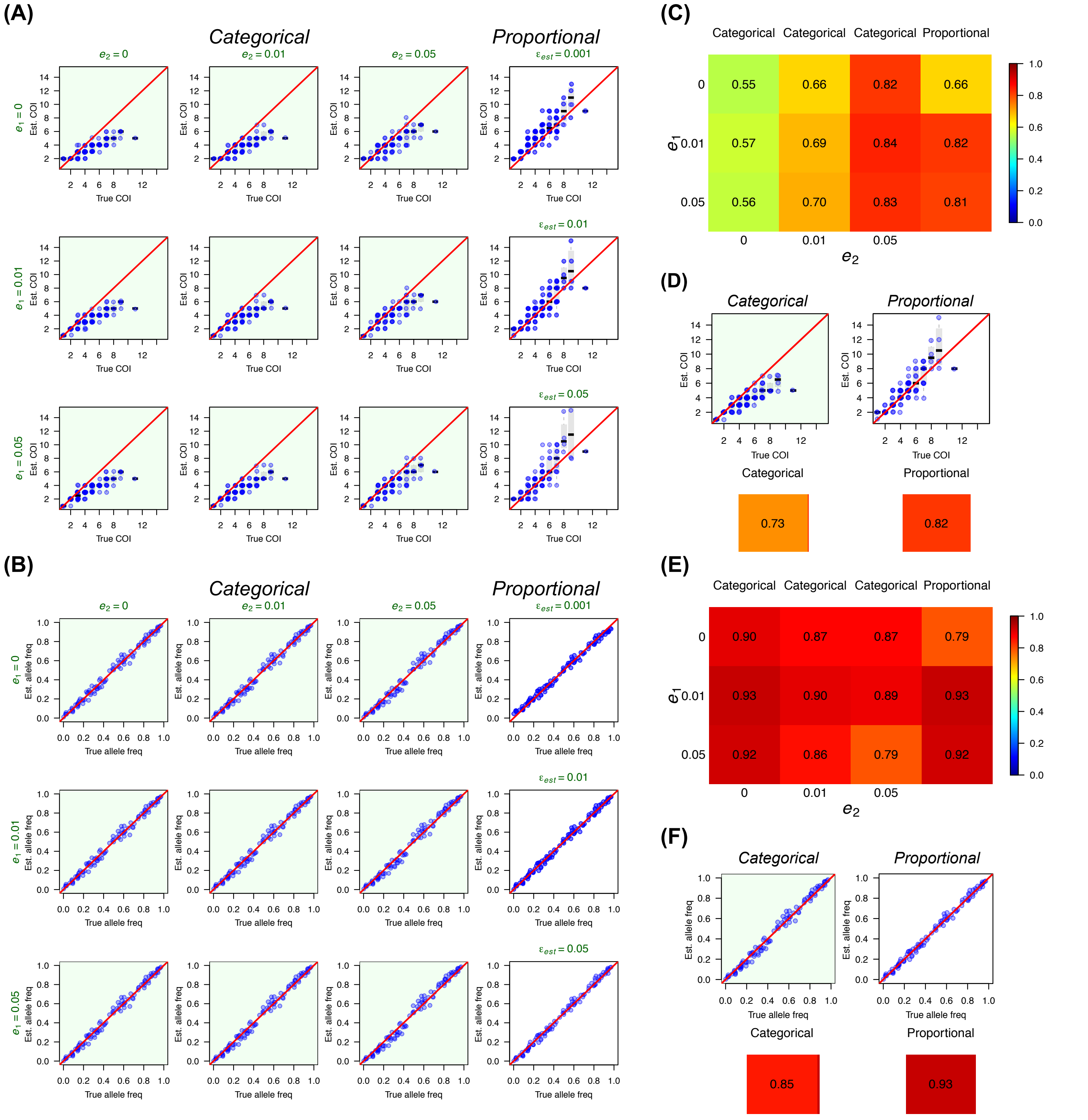

Fig C. Estimates of COI and allele frequencies using *THE REAL McCOIL* when simulations included measurement error. ε=0.01 and $\bar{m}$=5 were used to simulate data. The thresholds (d_1, d_2)=(5,85) were used for calling heterozygous/homozygous, and in this condition, the true e_1 and e_2 were 0.0086 and 0.099 in the simulated data. **(A)** True vs. estimated COI by categorical method and proportional method when parameters of measurement error used in the estimation procedure varied (e_1 and e_2 for categorical method [left three columns] and ε_{est} for proportional method [right column]). **(B)** True vs. estimated allele frequencies by categorical method and proportional method when parameters of measurement error used in the estimation procedure varied. **(C)(E)** The probability that 95% credible interval contained true COI (C) or allele frequencies (E) when parameters of measurement error used in the estimation procedure varied (the layout is the same as (A) and (B)). **(D)(F)** The comparison between true vs. estimated COI (D)

and allele frequencies (F) and the probability that 95% credible interval contained true value when parameters of measurement error were fitted as part of the MCMC. The probability that the 95% credible interval contained the true COI when parameters of measurement error were fitted (D) was higher than those when parameters of measurement error were greatly mis-specified (C). The 95% credible interval of ε_{est} contained true ε, while the 95% credible intervals of e_1 and e_2 did not contain true e_1 and e_2.

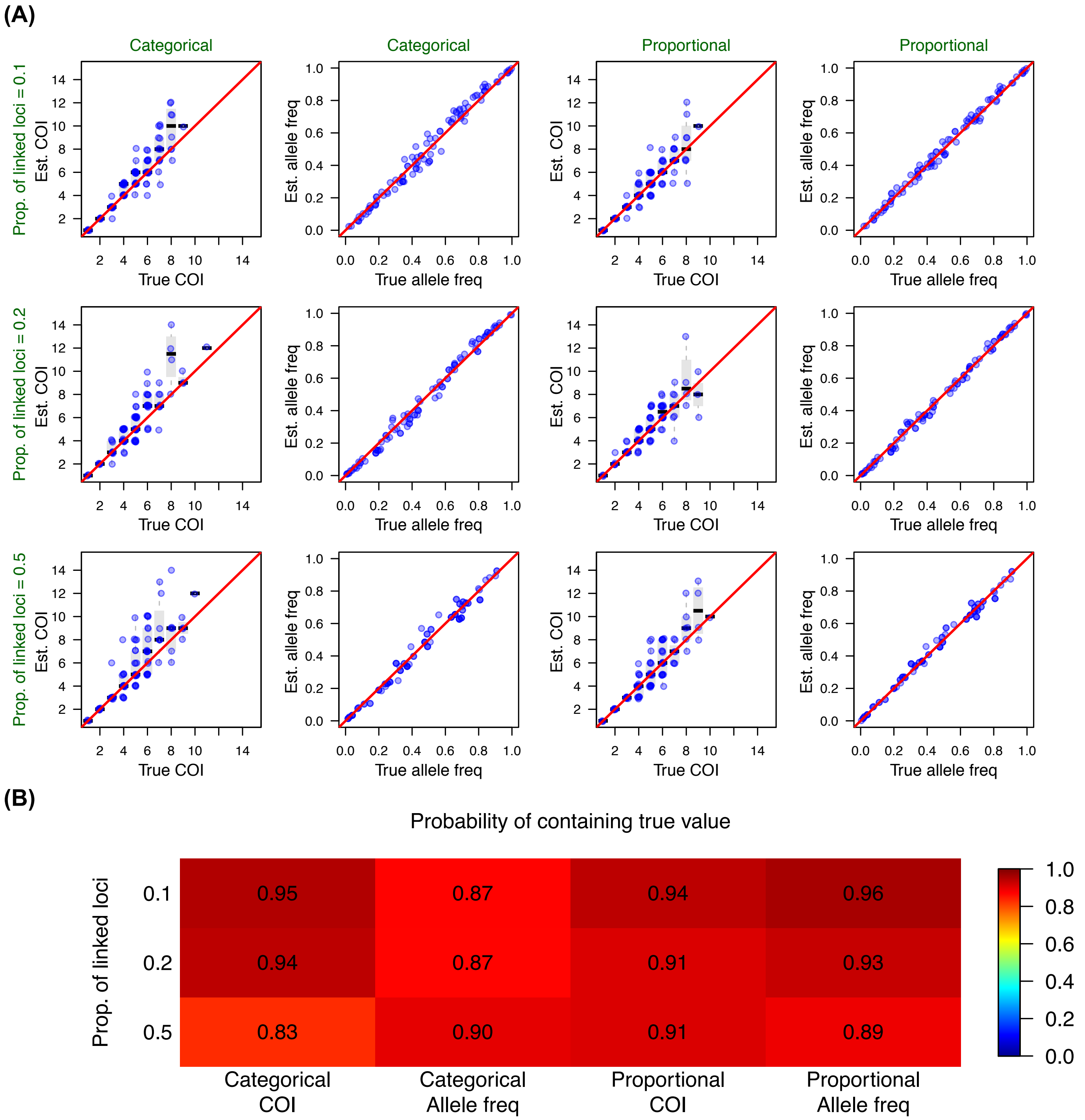

Fig D. The performance of *THE REAL McCOIL* when some of the loci were linked. (A) True vs. estimated COI and allele frequencies. **(B)** The probability that the 95% credible interval contained the true value.

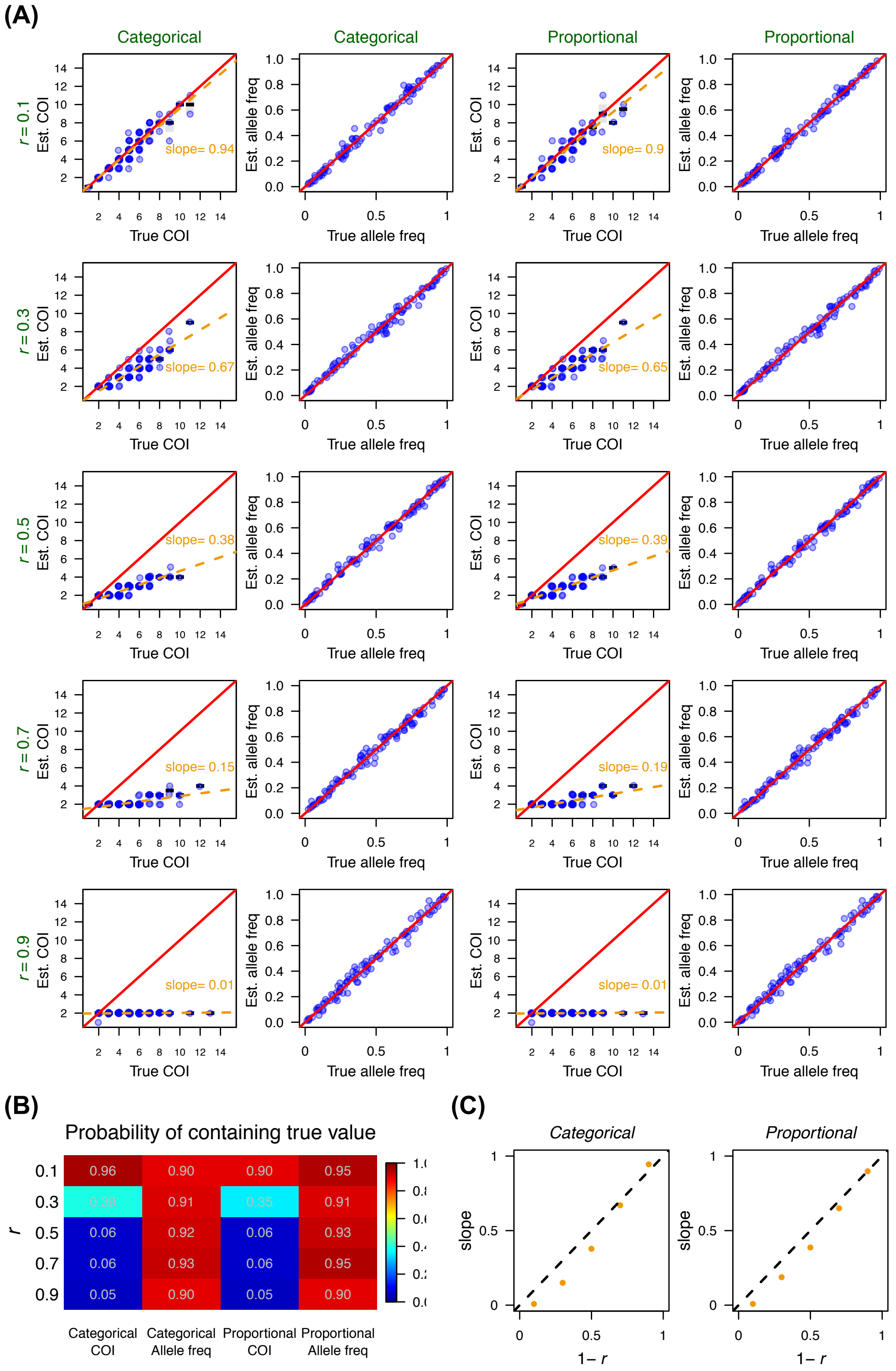

Fig E. The performance of *THE REAL McCOIL* when lineages within the same host were related. (A) True vs. estimated COI and allele frequencies. The slope and intercept of the red line are 1 and 0, respectively. The orange line was obtained by linear regression between estimated and true values of COI and the slope is shown in orange. **(B)** The probability that the 95% credible interval contained the true

value. **(C)** The comparison between the relatedness and the slope estimated from linear regression between estimated and true values of COI shown in (A).

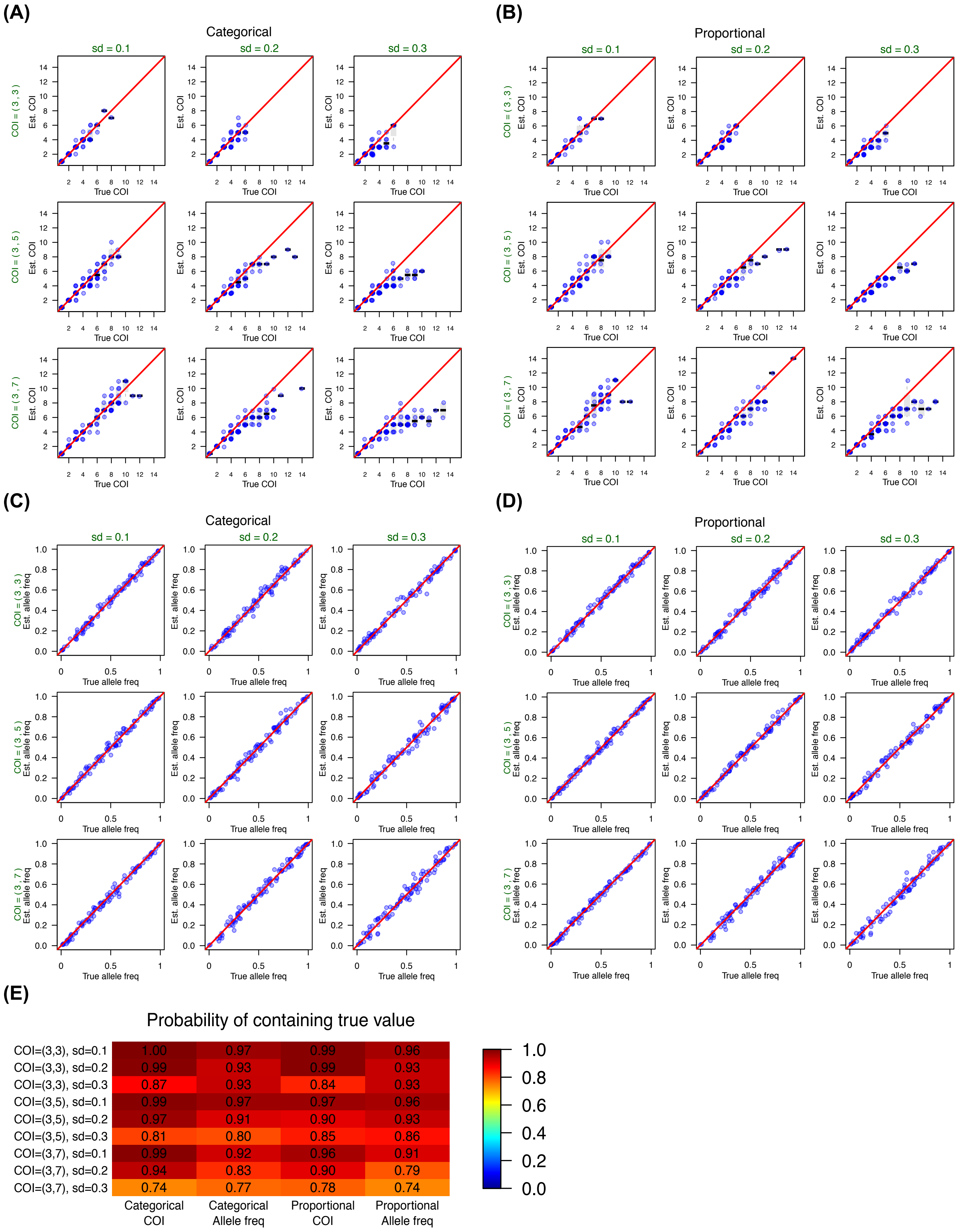
(A)
Categorical
sd = 0.1
sd = 0.2
sd = 0.3
COI = (3 , 3)
COI = (3 , 5)
COI = (3 , 7)
Est. COI
True COI
(B)
Proportional
sd = 0.1
sd = 0.2
sd = 0.3
COI = (3 , 3)
COI = (3 , 5)
COI = (3 , 7)
Est. COI
True COI
(C)
Categorical
sd = 0.1
sd = 0.2
sd = 0.3
COI = (3 , 3)
COI = (3 , 5)
COI = (3 , 7)
Est. allele freq
True allele freq
(D)
Proportional
sd = 0.1
sd = 0.2
sd = 0.3
COI = (3 , 3)
COI = (3 , 5)
COI = (3 , 7)
Est. allele freq
True allele freq
(E)
Probability of containing true value
COI=(3,3), sd=0.1 1.00 0.97 0.99 0.96
COI=(3,3), sd=0.2 0.99 0.93 0.99 0.93
COI=(3,3), sd=0.3 0.87 0.93 0.84 0.93
COI=(3,5), sd=0.1 0.99 0.97 0.97 0.96
COI=(3,5), sd=0.2 0.97 0.91 0.90 0.93
COI=(3,5), sd=0.3 0.81 0.80 0.85 0.86
COI=(3,7), sd=0.1 0.99 0.92 0.96 0.91
COI=(3,7), sd=0.2 0.94 0.83 0.90 0.79
COI=(3,7), sd=0.3 0.74 0.77 0.78 0.74
Categorical COI
Categorical Allele freq
Proportional COI
Proportional Allele freq
1.0
0.8
0.6
0.4
0.2
0.0

Fig F. The performance of *THE REAL McCOIL* when the population was not well mixed. We simulated two subpopulations with same or different average COI and with different allele frequencies. The difference in allele frequency was sampled from a normal distribution with mean=0 and variance = sd^2. **(A)(B)** True vs. estimated COI by categorical method (A) and proportional method (B). **(C)(D)** True vs. estimated allele frequencies by categorical method (C) and proportional method (D). **(E)** The probability that the 95% credible interval contained the true value. Estimation of allele frequency was robust, but COI was underestimated. The level of underestimation of COI increased with the difference in the average of COI and the difference in allele frequencies between two populations.

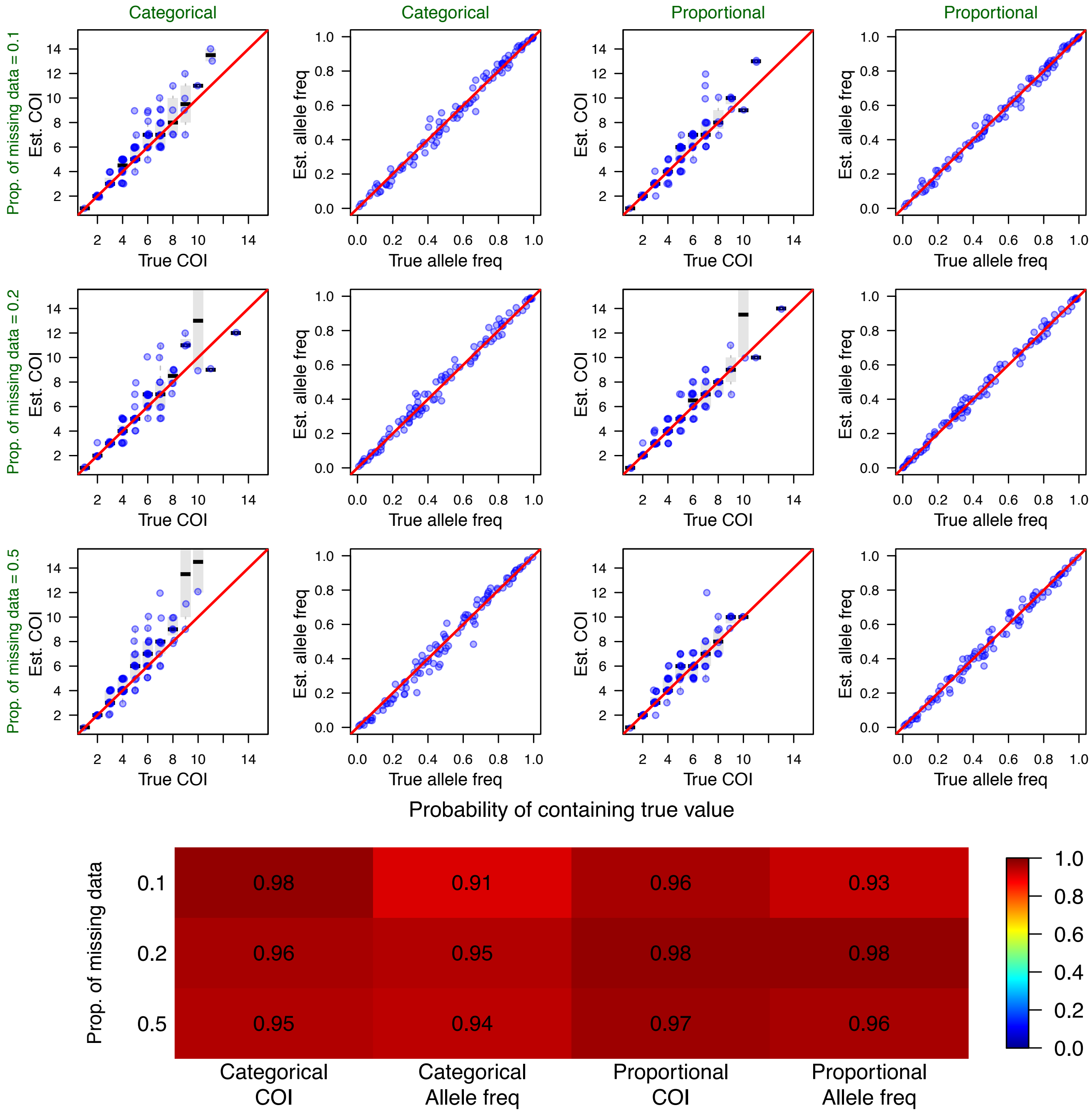

Fig G. The performance of *THE REAL McCOIL* when there were missing data. (A) True vs. estimated COI and allele frequencies. **(B)** The probability that the 95% credible interval contained the true value.

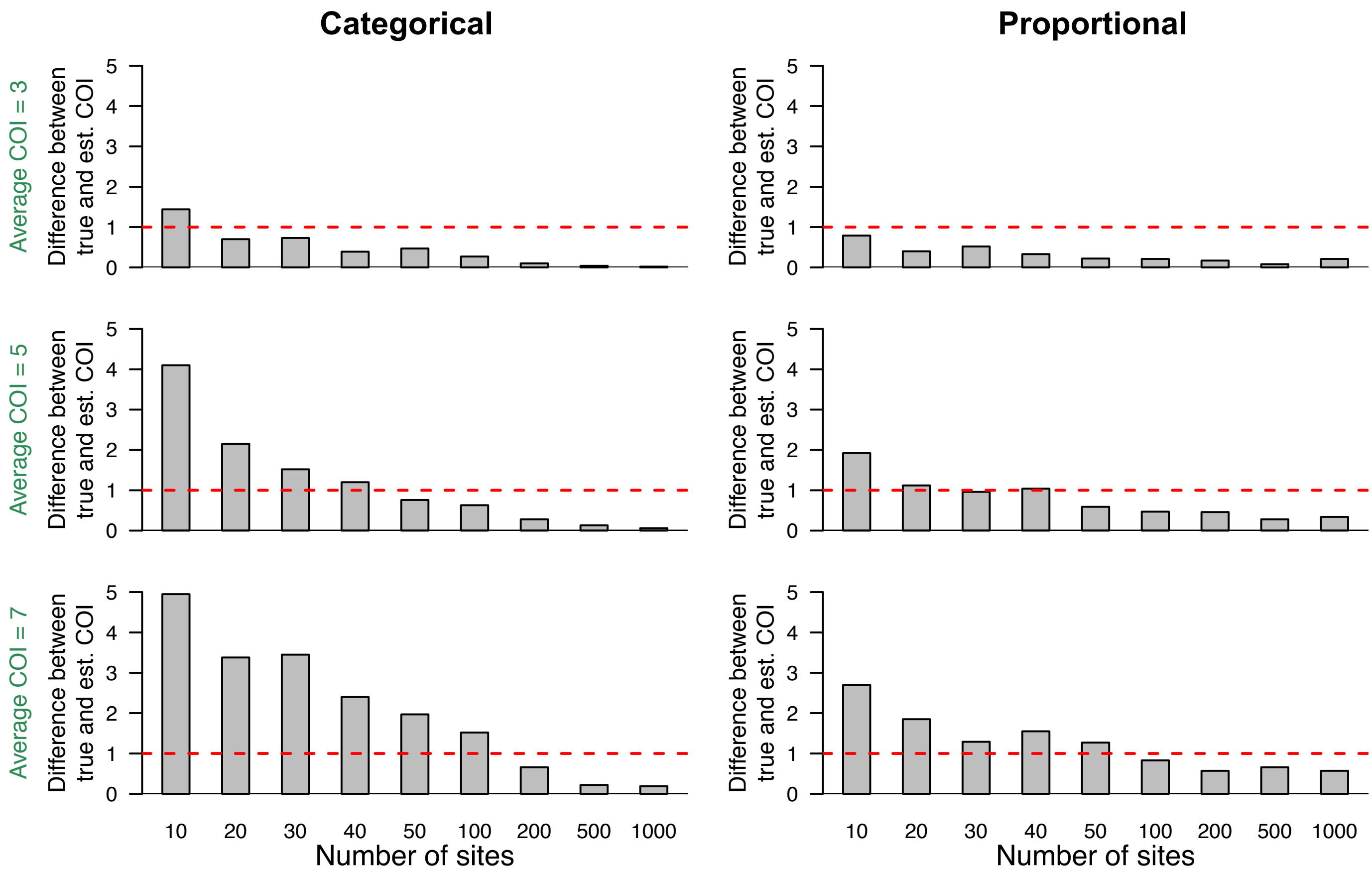

Fig H. The average difference between true and estimated COI decreased with the number of loci included in the analysis. The probability that 95% credible interval contained true COI was 1 for all cases here.

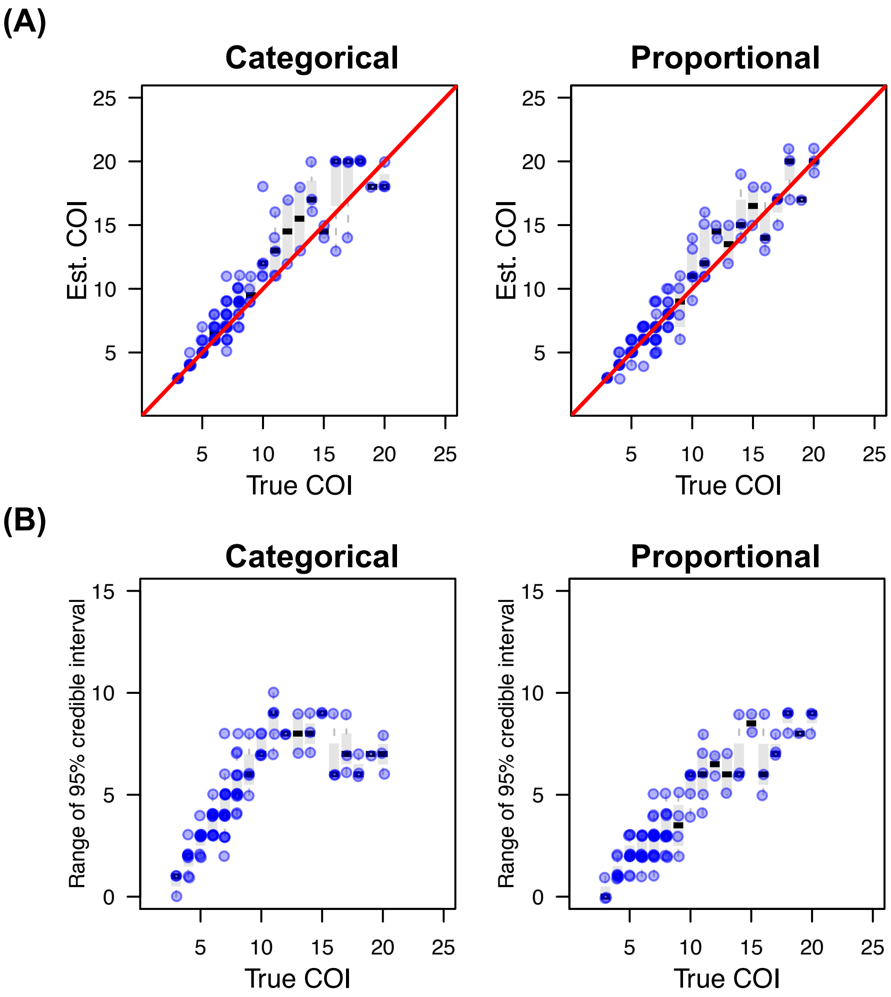

Fig I. (A) True vs. estimated values of COI using *THE REAL McCOIL* when COI is above 15. Each blue dot represents a sample. The black bar and the grey box show the median and 25% to 75% quantile. m_{max} =25 was used. **(B)** The range of 95% credible interval increased with true COI.

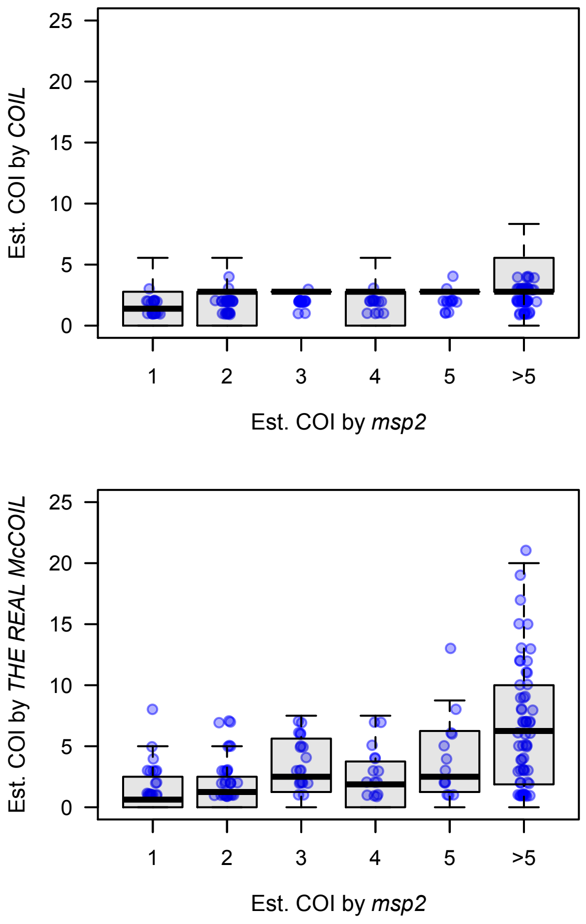

Fig J. Comparison between COI estimated from *COIL*, *THE REAL McCOIL*, and *msp2* typing. The estimated COI from *msp2* typing and those estimated from SNP data are significantly correlated, and the correlation between COI estimated from *THE REAL McCOIL* and *msp2* is larger than that between *COIL* and *msp2* (Pearson's correlation test, ρ=0.35 [*COIL*] and 0.45 [*THE REAL McCOIL*]; *p*-values= 1.1×10^{-5} [*COIL*] and 6.0×10^{-9} [*THE REAL McCOIL*]). e_1=e_2=0.05 were used in both *COIL* and *THE REAL McCOIL*.

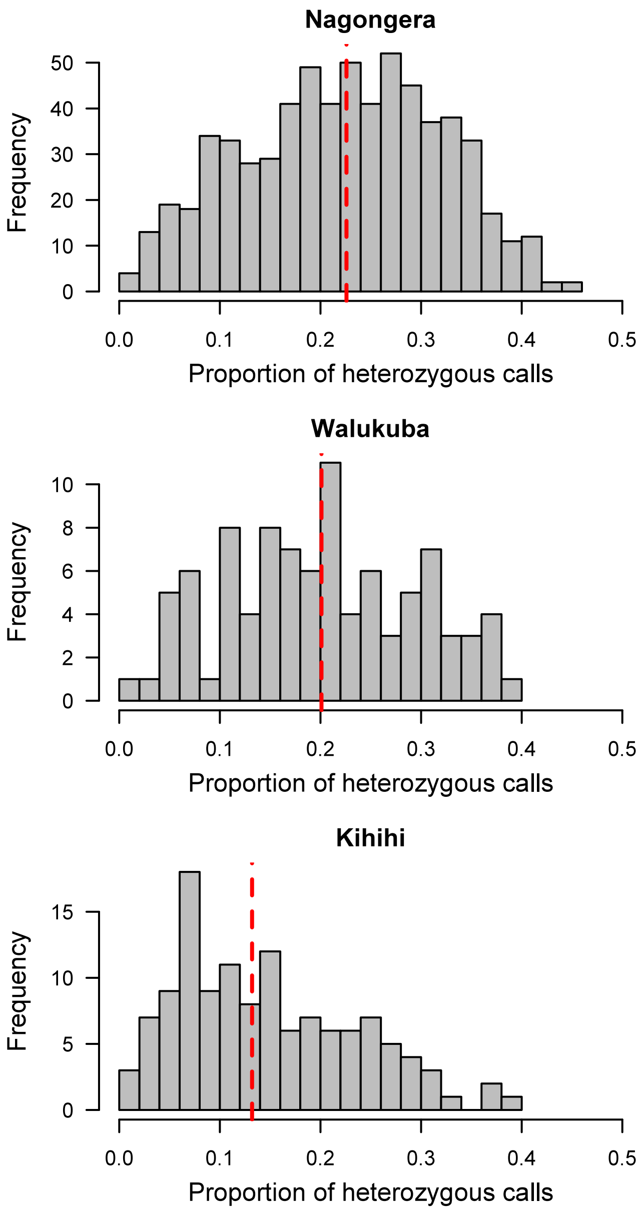

Fig K. The proportion of heterozygous calls of samples from in Nagongera, Walukuba, and Kihihi.

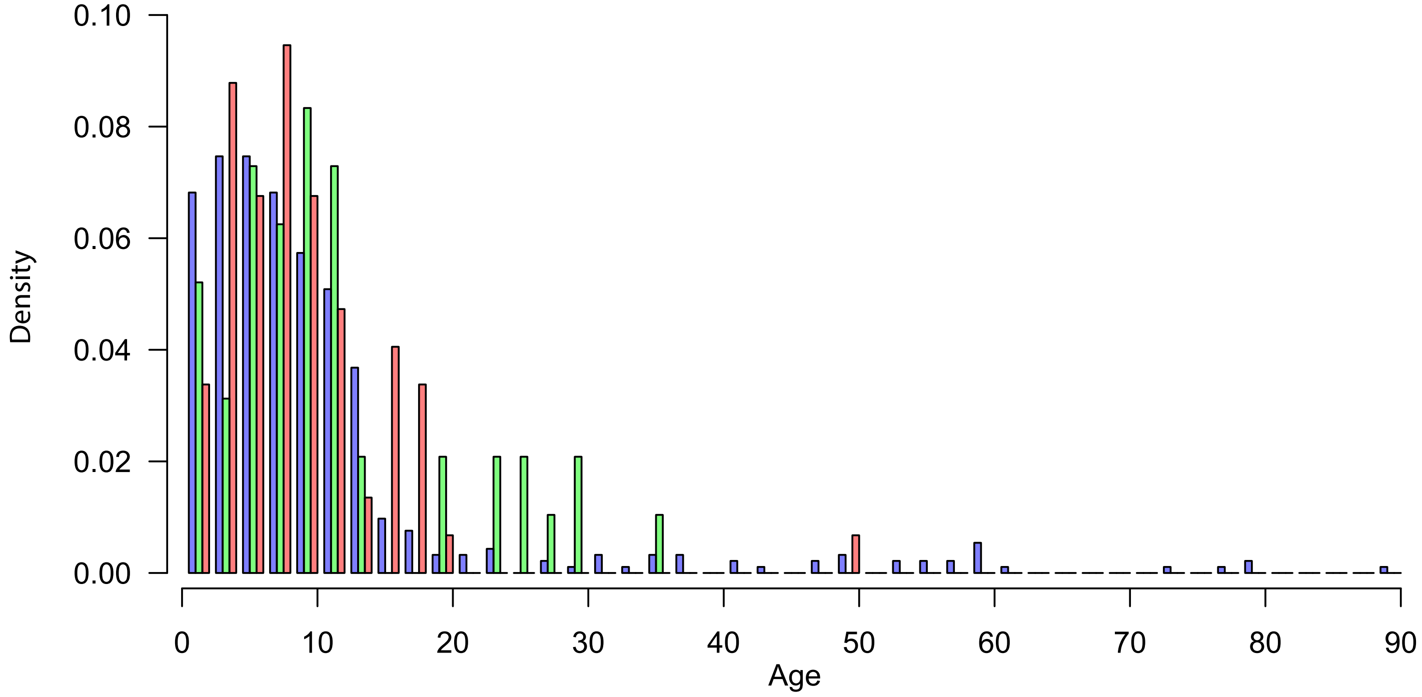

Fig L. The distributions of age of individuals with genotyped samples from Nagongera, Walukuba, and Kihihi.

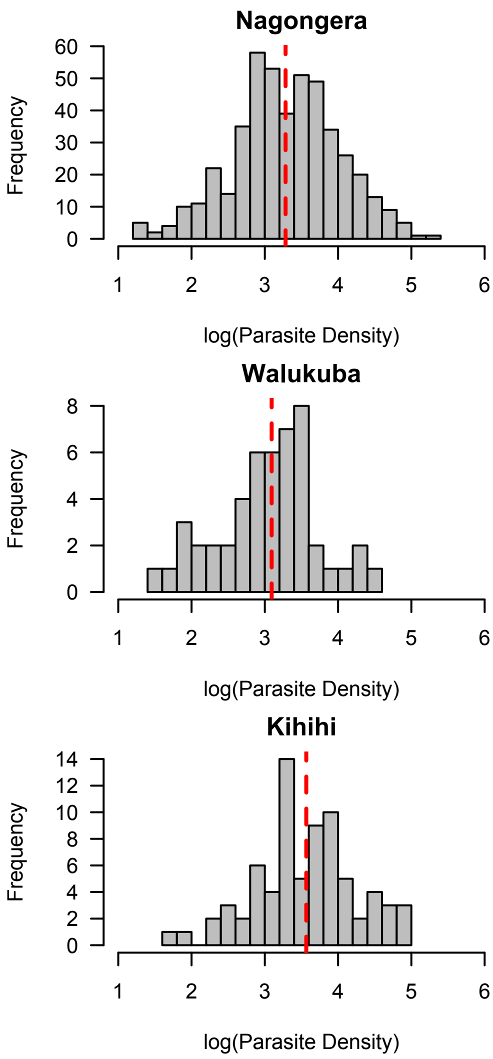

Fig M. The distributions of parasite density of samples from Nagongera, Walukuba, and Kihihi.

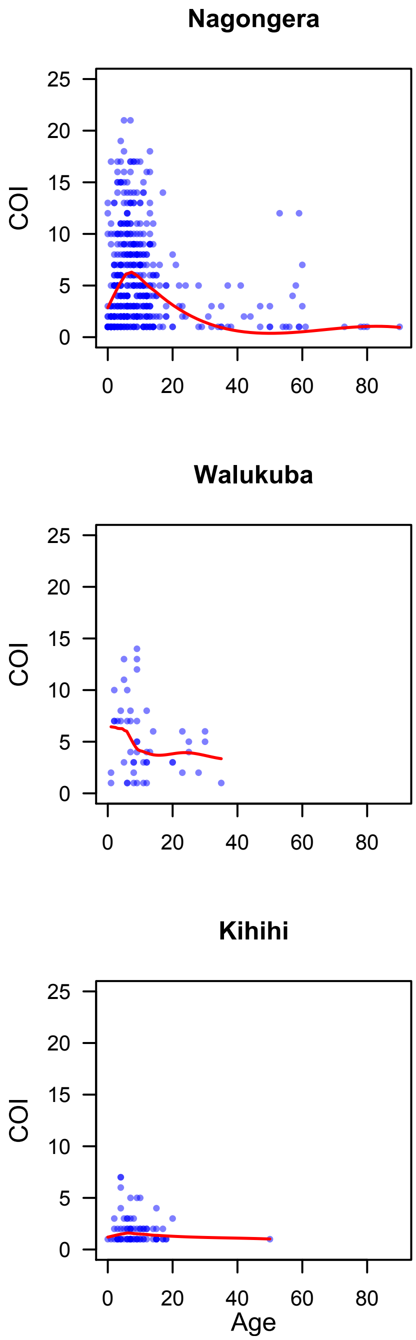

Fig N. The relationship between COI and age. COI increased with age until age 7, and decreased with age when it is above 7 in Nagongera. The sample sizes for Walukuba and Kihihi were small and the relationship between COI and age was less clear. The red lines are smooth curves computed by locally weighted scatterplot smoothing (loess).

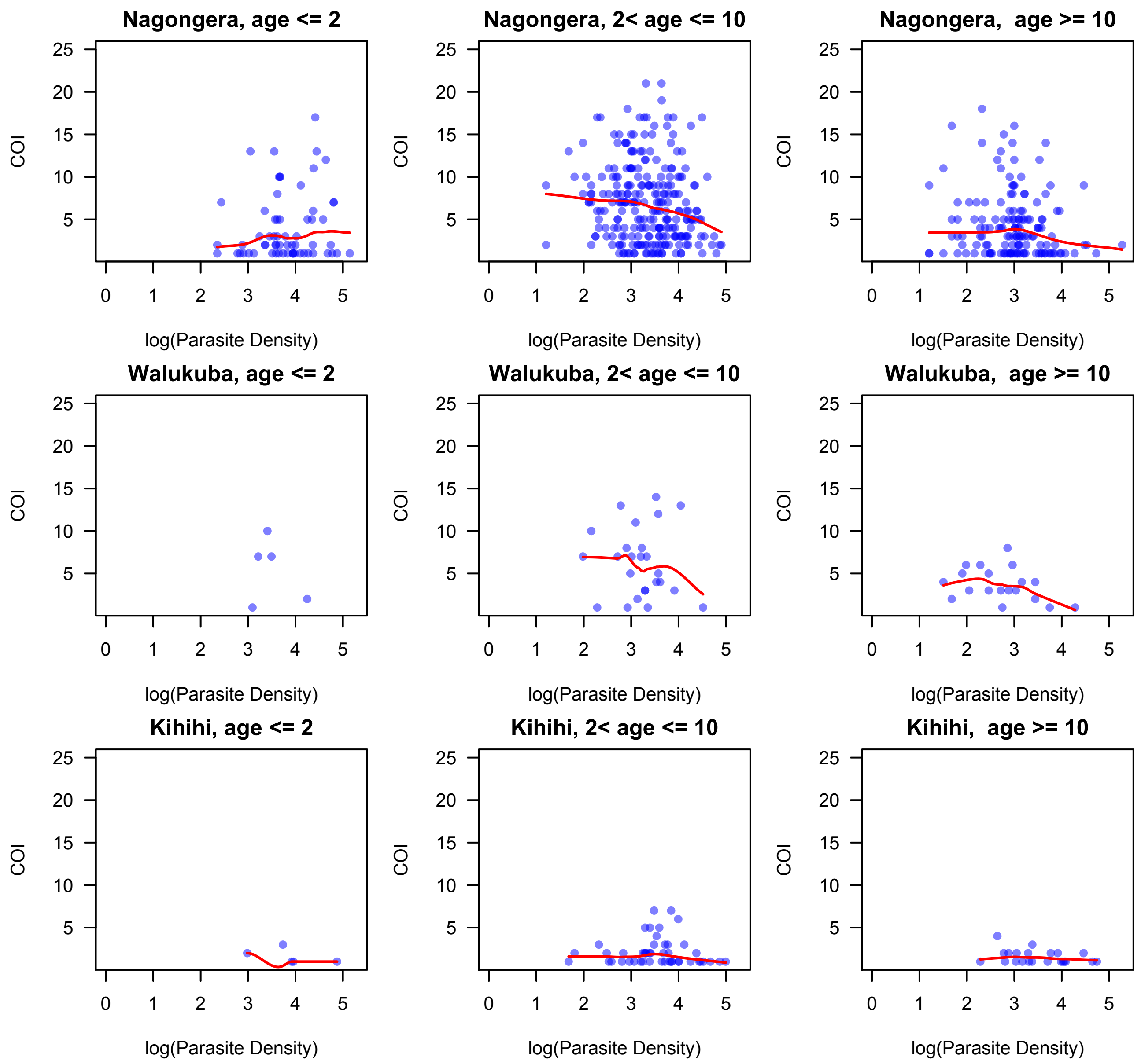

Fig O. The relationship between parasite density and COI. Parasite density was negatively correlated with COI after adjusting for age (partial correlation *r*= −0.15 [Nagongera], −0.27 [Walukuba], −0.23 [Kihihi], *p*-values= 0.0011 [Nagongera], 0.058 [Walukuba], 0.043 [Kihihi]). This negative association was most pronounced in those aged 3−10 years in Nagongera. The red lines are smooth curves computed by locally weighted scatterplot smoothing (loess).

Table A. List of parameters

Notation	Meaning	Default value
For simulation		
n	The number of samples	100
k	The number of loci	100
$\overline{m}$	Average COI (COI~ truncated Poisson($\overline{m}$))	5
$\overline{I}$	Average intensity of signal in the SNP assay	8
ε	Measurement error of signal in the SNP assay	0
x	The proportion of missing data	0
r	Relatedness (the probability that one allele is sampled from another lineage within the same host)	0
p	The proportion of loci that are linked with another loci	0
For analysis		
m_{max}	Upper bound for COI	20 for simulated data; 25 for Uganda data
N	The total number of MCMC iterations	100,000 for simulated data; 500,000 for Uganda data
	Heterozygous/homozygous data	
e_1	The probability of calling homozygous loci heterozygous	0.05 for Uganda data; 0 when ε =0; otherwise specified in the text
e_2	The probability of calling heterozygous loci homozygous	0.05 for Uganda data; 0 when ε =0; otherwise specified in the text
d_1, d_2	(d_1, d_2) is the range of degree to call heterozygous	(5,85) when ε >0; otherwise (0,90)
I_{min}	The intensity of signal needs to be greater than I_{min} to be considered in the analysis	1 when ε >0; otherwise 0
	Frequency data	
ε_{est}	Measurement error of signal in the SNP assay used during the estimation	0.02 for Uganda data; 0 when ε =0; otherwise specified in the text

Table B. Estimates of COI when parameters of measurement error used in the estimation varies

		Nagongera	Walukuba	Kihihi
**Categorical method**	**(e_1, e_2)**			
	(0.05, 0.05)	*5.77 (5)	5.23 (4.5)	1.93 (1)
	(0.01,0.05)	5.32 (4)	5.04 (4.5)	2.07 (2)
	(0.05,0.01)	4.43 (3)	4.50 (4)	1.86 (1)
	(0.01,0.01)	4.21 (3)	4.23 (4)	1.99 (2)
	(0, 0.05)	5.00 (4)	4.92 (4)	2.34 (2)
	(0, 0.1)	6.35 (5)	5.83 (5.5)	2.50 (2)
	(0, 0.2)	9.68 (8)	7.69 (8)	2.97 (2)
	estimated	7.30 (6)	5.25 (5)	2.59 (2)
**Proportional method**	ε_{est}			
	0.01	7.65 (6)	8.04 (7.5)	3.43 (2)
	0.02	7.33 (5)	7.81 (7)	3.20 (2)
	0.05	7.40 (4)	8.13 (7)	2.99 (2)
	estimated	7.39 (5)	7.83 (7)	3.01 (2)

* Both mean (outside of bracket) and median (in bracket) of COI in three locations are shown.

Table C. Pairwise F_{ST} among Nagongera, Walukuba, and Kihihi

	Walukuba	Kihihi
**Nagongera**	0.008 (-0.009)*	0.04 (0.006)
**Walukuba**		0.004 (-0.007)

*The estimates of F_{ST} using allele frequencies estimated from categorical method (outside of bracket) and proportional method (in bracket).
